# Mapping cancer biology in space: applications and perspectives on spatial omics for oncology

**DOI:** 10.1186/s12943-024-01941-z

**Published:** 2024-01-30

**Authors:** Sumin Lee, Gyeongjun Kim, JinYoung Lee, Amos C. Lee, Sunghoon Kwon

**Affiliations:** 1 Department of Electrical and Computer Engineering, Seoul National University, Seoul, 08826 Republic of Korea; 2 Meteor Biotech,, Co. Ltd, Seoul, 08826 Republic of Korea; 3 Interdisciplinary Program in Bioengineering, Seoul National University, Seoul, 08826 Republic of Korea; 4 Division of Engineering Science, University of Toronto, Toronto, Ontario, ON M5S 3H6 Canada; 5 Bio-MAX Institute, Seoul National University, Seoul, 08826 Republic of Korea; 6 Institutes of Entrepreneurial BioConvergence, Seoul National University, Seoul, 08826 Republic of Korea; 7 Cancer Research Institute, Seoul National University College of Medicine, Seoul, 03080 Republic of Korea

## Abstract

Technologies to decipher cellular biology, such as bulk sequencing technologies and single-cell sequencing technologies, have greatly assisted novel findings in tumor biology. Recent findings in tumor biology suggest that tumors construct architectures that influence the underlying cancerous mechanisms. Increasing research has reported novel techniques to map the tissue in a spatial context or targeted sampling-based characterization and has introduced such technologies to solve oncology regarding tumor heterogeneity, tumor microenvironment, and spatially located biomarkers. In this study, we address spatial technologies that can delineate the omics profile in a spatial context, novel findings discovered via spatial technologies in oncology, and suggest perspectives regarding therapeutic approaches and further technological developments.

## Introduction

Cancer research has a long history dating back to ancient times, with our understanding of cancer growing as technology advances [1]. Especially, microscopic observations and staining methods have made significant progress in cancer research [2]. Microscopic observations have led to the discovery of fundamental principles in tumor biology, such as the identification of cells as the basic unit of organisms, the abnormal dividing phenomenon of cancer cells, the morphological distinction of carcinoma subtypes, and the discovery of cancer cells originating from normal cells [2–5]. To better distinguish cells, Joseph von Gerlach first stained tissues with carmine [5], which eventually led to the discovery of hematoxylin and eosin (H&E) staining [6], a staining method widely used these days to examine the overall cellular organization [7]. Immunostaining and hybridization-based staining methods have been developed to detect antigenic heterogeneity, biomarker discovery, therapeutic prognosis, spatial heterogeneity, etc. [8–15].

In addition to the visual examination of cancer tissues, advanced methods to analyze the cellular composition of cancer have been developed [16]. The Human Genome Project outputted the full human genome, critically based on the belief that cancer cells derived from normal cells due to gene mutations, thus serving as a code that can be compared to obtain cancer-related gene sequences [17, 18]. In 2005, the Cancer Genome Atlas Project was launched and completed successfully by 2018, which constructed a comprehensive atlas of cancer-related genes for further research [19]. Cellular heterogeneity has been recognized in cancer research, followed by the development of single-cell sorting and sequencing technologies [20–22] to decipher intratumoral heterogeneity [23], tumor progression [24, 25], and metastasis.

As described, the development of technology involves novel questions that have not been unraveled before. Tumors are not simply a group of malignant cells; rather, they construct the tumor architecture resulting in the underlying cancerous cellular mechanisms [26]. Increasing research has reported the importance of the spatial context of tumor architectures for resolving the mechanisms of tumor initiation, progression, metastasis, and therapeutic response and so on. Tumor cells interact with the microenvironment nearby building the tumor immune microenvironment of immune cells, such as macrophages, B-cells, T-cells, and dendritic cells, indicating the reasons for immune reactions in tumor architectures [24, 27–31]. For instance, spatial analysis of tissue architectures has been actively employed to uncover unique tumor architectures such as Tertiary Lymphoid Structures (TLS), which are immune cell-rich structures which can indicate the ongoing anti-tumor immune response [32]. Moreover, drug resistance and cancer therapeutic strategies are significantly affected by the spatial distribution of tumors, emphasizing the need for the novel spatial discovery of therapeutic biomarkers [31, 33–35]. Clinical outcomes depend on the spatial composition of the cancer subtypes [36–38]. Therefore, spatial analysis of tissue architectures has recently been actively conducted to decipher tumor heterogeneity and tumor microenvironment, and to identify novel biomarkers.

**Fig. 1:**
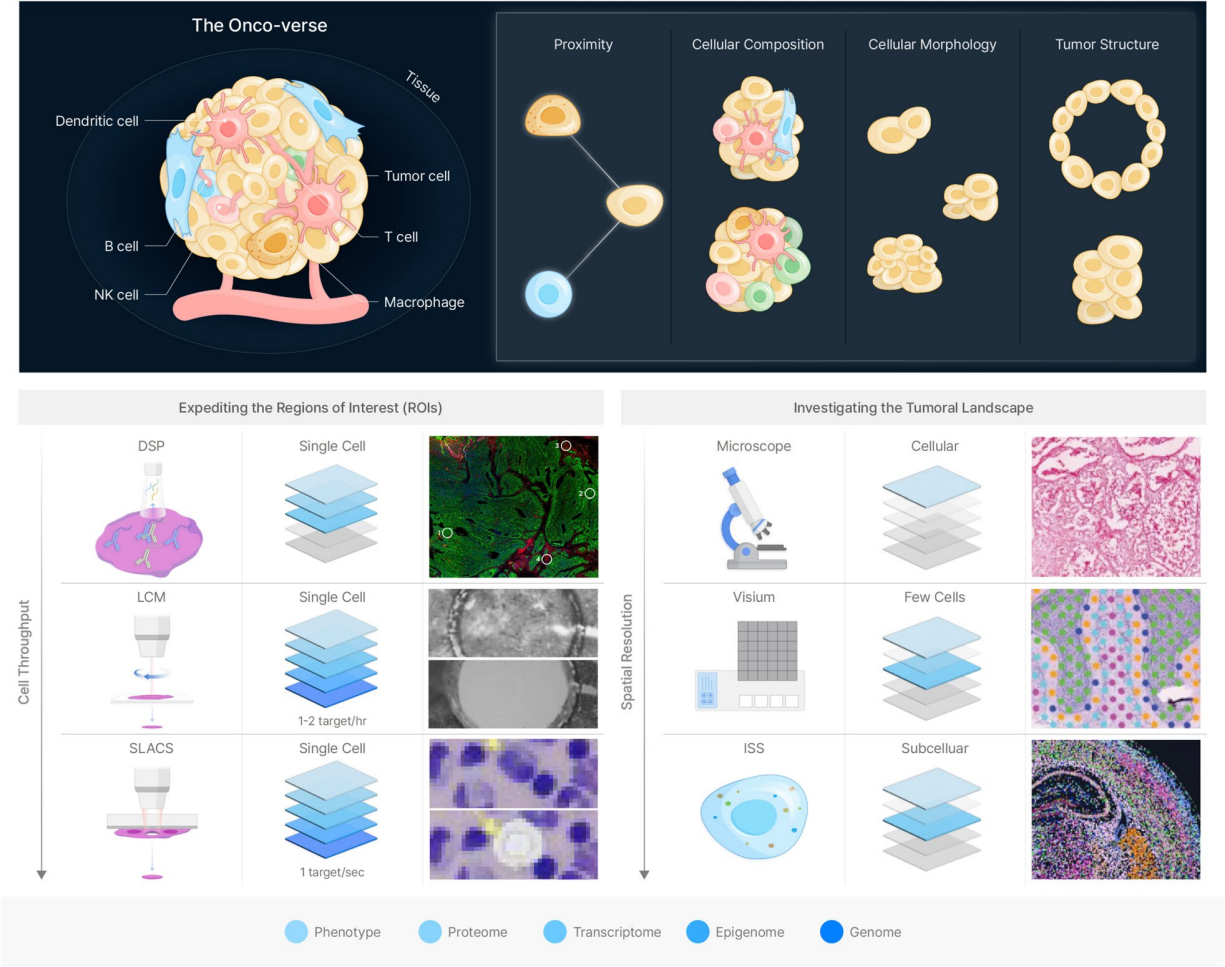
Mapping onco-verse in spatial context

Spatial analysis in tumor research can provide principle information regarding proximity, cellular composition, morphology and structure [39–43] (Fig. 1). Proximity refers to the physical distance between cells, which can be important for understanding cell-cell interactions and the distribution of different cell types within a tumor [44–46]. Cellular composition refers to the types of cells that make up a tumor, including cancer cells, immune cells, and stromal cells [47, 48]. Analyzing the spatial distribution of different cell types can provide insight into the tumor microenvironment and potential therapeutic targets. Morphology refers to the shape and size of cells within the tumor, which can be important for distinguishing different cell types and understanding their functions [26, 49, 50]. Structure refers to the overall organization of cells within the tumor, including the presence of different regions or zones within the tumor [51, 52]. Analyzing the spatial structure of a tumor can provide insight into the growth and progression of the tumor. By considering these components in the analysis of the spatial context of a tumor, researchers can gain a more comprehensive understanding of the tumor microenvironment, potential therapeutic targets, and the mechanisms driving tumor growth and progression.

A number of spatial omics technologies have been developed recently, and are being applied to various cancer researches [53]. They can be categorized into spacecraft-like technology, which deciphers in-depth characterizations of contextually important regions and telescope-like technology, which delineates the overall landscape of the tumor architecture. Spacecraft-like technology, such as targeted sampling or ROI (region-of-interest) profiling technologies, allows researchers to dive deeper and analyze in-depth characterizations of contextually important regions of the tumor [54–57]. Targeted sampling technologies allow researchers to analyze specific subpopulations of cells within the tumor and identify molecular changes that are unique to these regions. This can be helpful for identifying potential therapeutic targets that are specific to certain subpopulations of cells within the tumor, and can provide a more detailed understanding of the underlying molecular mechanisms driving tumor growth and progression. In contrast, telescope-like technologies, provides a broad view of the molecular landscape of the tumor architecture [58–64]. These spatial landscaping technologies allow researchers to observe the overall gene expression and protein localization patterns in a tumor sample. This can be helpful for identifying general trends and patterns in the molecular biology of the tumor, including changes in the tumor microenvironment, and can provide a global view of the tumor architecture.

This review introduces technologies that were used or are currently being used to address the spatial context in tumors, presents novel findings regarding spatial omics, and discusses future perspectives for novel therapeutic strategies. The scope of this review is to examine two distinct types of technologies - targeted sampling technologies and spatial landscaping technologies - and to explore their applications and future perspectives in the field of cancer research. There are comprehensive review papers that describe and compare spatial omics technologies [65–68] to help understand what is discussed in this review.

### Main

#### LCM: spacecraft for analyzing the tumor region of interest

**Fig. 2:**
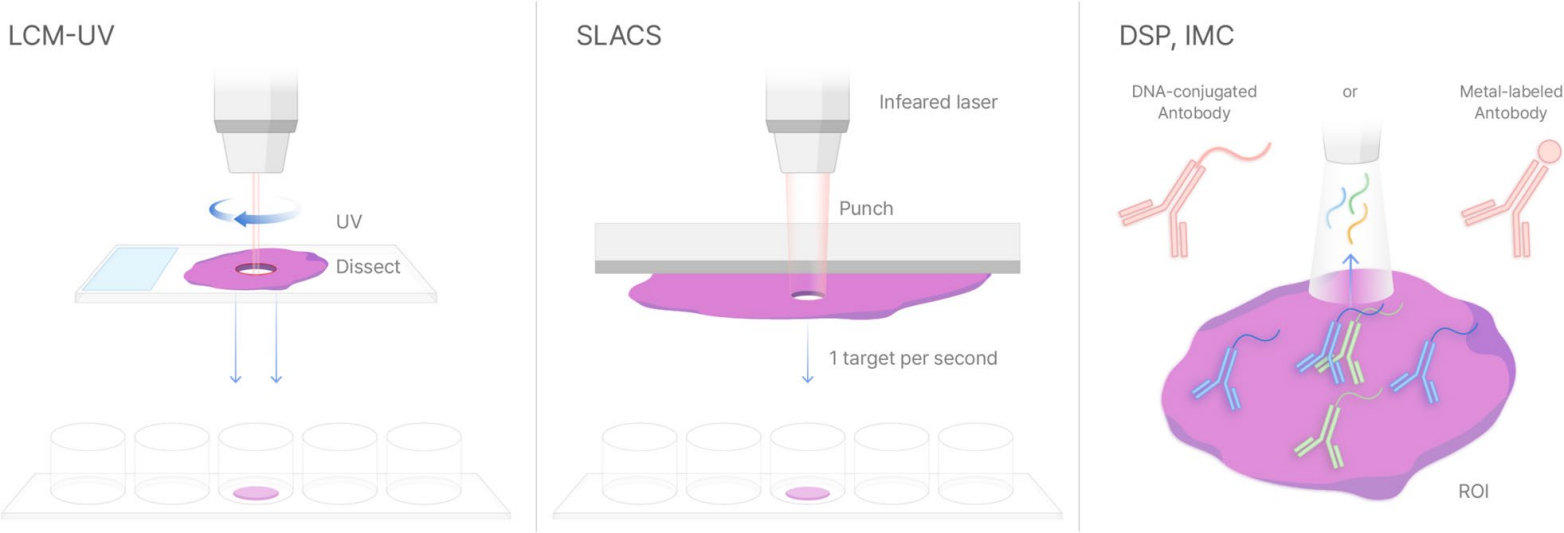
Targeted sampling-based spatial omics profiling technologies

FACS (fluorescence-activated cell sorting) has been a highly versatile technique to isolate cells from heterogeneous populations based on their physical and chemical characteristics. One of the main advantages of FACS is that it can be applied to a wide range of post-processing assays. As the cells of interest are sorted out, they can be subjected to various assays to profile its gene expression, protein expression and other functional assays. However, FACS requires cells to be prepared in suspension, which results in the loss of spatial information about the cells. There are technologies similar to FACS that can retrieve cells of interest and apply various chemistries while preserving their spatial position. Just as spacecrafts focus on observing specific celestial bodies to gather deeper information, these technologies focus on regions of interest to provide deeper genetic information in those regions (Fig. 2).

Needle biopsy is the initial form of spacecraft used in cancer research to physically extract cancer cells from different spatial contexts [36, 69]. For the precise selection of the specific regions, Laser Capture Microdissection (LCM) was introduced in the 1990s by Emmert-Buck [54], and has been used to isolate the region of interest in tumor tissue to integrate molecular analysis of the genome, epi-genome transcriptome, proteome, metabolome and multi-omics [56, 70]. LCM performs isolation using two main technologies: one using an IR laser to melt the EVA polymer attaching the region of interest (ROI) and another using a UV laser beam dissecting out the ROI section [71]. LCM can isolate a low homogeneous population from heterogeneous tumor tissue, thereby extracting a single cell or subcellular tissue in a rapid manner. Owing to its ability to easily isolate the ROI from the entire tissue, LCM has provided detailed insights into cancer. In the early stages, LCM was used to check for loss of heterogeneity, DNA genotyping, gene expression analysis, signal-pathway analysis, and protein analysis [70, 72]. LCM is currently being applied in cancer research, yielding a spatial modality in cancer omics for therapeutic and diagnostic purposes.

Combined with conventional DNA sequencing technologies, LCM has been used to resolve the lineage of cancer subclones or search for genomic mutations, such as single nucleotide variants (SNV) or copy number variations (CNV), in heterogeneous tumor populations. Using mate-pair sequencing, CNV was detected to draw a phylogenic relationship with other tumors and histopathological types in Testicular Germ Cell Tumors (TGCTs) as well as Structural Variants (SV), which can become a neoantigen in cancer therapeutics [73]. The protocol for whole-genome amplification in low-input genome samples was developed using LCM to investigate somatic mutations in non-neoplastic tissues [74]. In addition, circulating tumor cells (CTCs) could also be studied using LCM by extracting a single cell in hydrogel encapsulated CTC to reveal genotyping and mutation features, since CTCs are rare in human blood and associated with metastasis in tumor patients [75].

Transcriptomic signatures were also deciphered in a spatial context using LCM. Following the explosive development of RNA sequencing technologies, differential gene expression has been thoroughly investigated after LCM dissection of spatially heterogeneous regions. Together with SMART3-seq, microniches of the epithelial cells in nasopharyngeal cancer and normal cells were compared, uncovering the activation of FGF and NF-B signaling pathways in tumor samples [76]. We were able to compare differences in gene expression between low Gleason grade, high Gleason grade, benign samples, and stroma samples from a single tissue block in prostate cancer to discover that stromal cells may induce metastatic progression [77]. Spatiotemporal analysis was performed in glioma to determine whether the COL1A1 gene affects the inhibition of tumor progression, which can be an actionable therapeutic target [78]. Through RNA sequencing of spatially distributed tumor samples, specific biomarkers or pathways related to tumor progression or metastasis can be identified.

Additionally, LCM-based spatially resolved proteomics has a significant advantage in that it allows spatial information to be added to mass spectrometry, the most comprehensive tool for quantitatively profiling proteins [79, 80]. A novel biomarker, methyltransferase nicotinamide N-methyltransferase expression in the stroma, which affects cancer-associated fibroblast differentiation, tumor progression, and metastasis, was discovered by analyzing differentially expressed proteins via LCM [81]. Another study conducted on lung cancers identified the characteristic proteomic compositions related to tumor progression [82]. Invadosome-related subcellular structural proteins have been identified, suggesting potential therapeutic biomarkers [83]. Compared with conventional spatial proteomics technologies, which have to design antibodies for target proteins, LCM-MS-based spatial profiling technology has the advantage of *de novo* spatial marker discovery.

Owing to recent developments in epigenetic assays, LCM can easily be expanded for spatial epigenome profiling. Changes in DNA methylation levels in terms of cell differentiation and organ development have been thoroughly investigated between adrenocortical tumor samples and adjacent normal samples using reduced representative bisulfite sequencing (RRBS) [84, 85]. In addition, methylation patterns at single base-pair resolution were found using LCM with whole-genome bisulfite sequencing (WGBS) in CTC in lung cancer [86]. The compatibility of LCM with other epigenetic chemistries such as ATAC-seq or Cut&Tag will expand biological findings regarding the spatial heterogeneity of epigenetic features in cancer research.

LCM has advantages of being compatible with the existing chemistry and capable of profiling two or more molecular targets. LCM-MS combined with RNA sequencing helped in better suggesting the reliable stratification of tumor subtypes which showed better relevance to survival rate in glioblastoma [87]. Moreover, by combining DNA sequencing and RNA sequencing in LCM-dissected spatial microniches, it was possible to distinguish three evolutionary pathways relevant to specific mRNA signatures with different survival outcomes in Triple Negative Breast Cancer (TNBC) [88]. Likewise, integrating existing molecular profiling techniques with spatially significant microniches provides better information for the characterization of tumor cells. The conventional LCM technique has low throughput and may induce cell damage; however, it is still a powerful tool to grant spatial modality to molecular features.

#### Novel cell sorting technologies: space probe for deeper analysis

In terms of probing different cells that exist within a spatial context, novel cell sorting technologies have emerged as useful tools for applications in cellular investigations for biomarker discovery. For example, intelligent image-activated cell sorting and Raman image-activated cell sorting have been developed for sorting cells according to their morphology and other image-based knowledge obtained using Raman imaging [89, 90]. Although these cell sorting methodologies require the cells to be dissociated into a solution state, they provide an efficient method to sort out cells labeled with cellular phenotypes. These sorting technologies enable single-cell level analysis of cells that cannot be labeled and sorted using conventional flow cytometry-based methodologies, such as fluorescence-activated cell sorting (FACS).

An advanced form of LCM has been reported, focusing on the spatial context of cells. In particular, Spatially resolved Laser-Activated Cell Sorting (SLACS) technology, which utilizes image-based information to sort cells without any loss of spatial information, has been used in several applications for studying cancer in a spatial context. SLACS is similar to LCM technologies; however, it does not have any dissection step and instead isolates cells with near-infrared laser pulses. The main advantage of SLACS lies in the versatility of the spatial and omics assays according to the user’s needs [91]. Conventional staining methodologies can guide the cells of interest or microniche of interest, and novel spatial technologies such as spatial transcriptomics or *in situ* sequencing technologies can guide the regions to be isolated. In addition, from an omics point of view, the retrieved cells of interest can undergo NGS-based assays or mass spectrometry-based assays. The first demonstration using SLACS was reported using breast cancer tissue sections and by analyzing different microniches using multiple displacement amplification to reveal the subclonality and evolutionary relationship between these different subclones [92, 93]. Using SLACS, 3D genomic maps of different subclones were analyzed using whole genome sequencing, whole exome sequencing, and targeted sequencing to construct and visualize the genomic landscape of breast cancer tissue sections. SLACS has also been applied to a circulating tumor cell (CTC) capturing biochip, where CTCs can be stained with immunofluorescence *in situ*. The CTCs of interest were then sorted with SLACS to perform whole genome sequencing [94]. SLACS has also been applied in spatial transcriptomics and epitranscriptomics in cancer biology. Lee et al. used SLACS to analyze different cell populations categorized as breast cancer stem cell markers ALDH1 and CD44 [55, 95]. Immunofluorescence label-guided SLACS showed full-length RNA sequencing of different microniches of interest to reveal a unique adenosine to inosine (A-to-I)-edited GPX4 gene in cancer stem cell microniches. They further showed that the discovered A-to-I-edited variant of GPX4 can be used as a predictive tool for triple-negative breast cancer patients who have received neoadjuvant chemotherapy. Probing the microniches of interest, SLACS provides a way to reveal specific markers that are unique to specific cell populations labeled with specific spatial assays (i.e., immunofluorescence in this case). In addition, Hema-seq, developed by Jeong et al., represents integration of cytopathological and genomic profiling, providing the understanding of complex hematological malignancies like simultaneous myeloma and acute myelogenous leukemia (AML) by mapping clonal changes within hematopoietic lineages [96]. This method combines whole-genome sequencing with detailed cytogenetic analysis, offering new insights into the molecular landscapes of blood tumors, albeit with the need for further validation across diverse hematological conditions. The potential for SLACS to be applied to genomics, transcriptomics, and proteomics in cancer biology can lead to the identification of specific diagnostic or therapeutic targets from certain populations that can be labeled with any spatial assay.

Furthermore, Nanostring have been actively developing its technology to spatially analyze RNA expression and protein abundance. Digital Spatial Profiling (DSP; Commercialized as GeoMx), a platform that profiles proteins and RNAs, was launched in 2019 [57]. The antibodies or target RNA complementary sequences, which have specific oligonucleotides attached to UV photocleavable linkers, were pooled on the tissue slide, revealing the spatial context within the tissue. Then, based on the user-defined region of interest (ROI), UV light exposure cleaves the UV photocleavable linker, freeing the oligonucleotide sequences that will be further collected and analyzed by either nCounter, which has 800-plex detection, or next-generation sequencing [97]. DSP is mainly used to study tumor heterogeneity, immune cell heterogeneity in the tumor microenvironment, and protein abundance during tumor-immune interaction [31, 43, 98, 99]. For example, DSP discovered an abundance of checkpoint protein CTLA4 surrounding pancreatic ductal adenocarcinoma, which supports tumors in avoiding the adaptive immune response caused by overexpressed genes [99].

Image Mass Cytometry (IMC) has emerged as a transformative technology in the spatial profiling of proteins (Commercialized as Hyperion) [100]. IMC utilizes heavy metal-labeled antibodies instead of traditional fluorophores to detect proteins in tissue sections. In IMC, after the laser ablates the tissue and releases the metal-labeled antibodies, these particles are ionized and then analyzed by the TOF mass cytometer. Each metal tag corresponds to a specific antibody (and therefore a specific protein), therefore identifying the metal tags allows researchers to determine which proteins were present in the tissue and where they were located. The great advantage of using metal tags and TOF mass cytometry in this context is that it allows for the simultaneous detection of many different proteins with high precision. In a similar vein, Multiplexed Ion Beam Imaging (MIBI) represents another leap forward in spatial proteomics combined with secondary ion mass spectrometry (Commercialized as MIBIscope) [101]. By focusing a primary ion beam on the sample, MIBI releases secondary ions from the metal tags, which are then analyzed to create detailed images of protein distribution. The precise quantification and localization of proteins at the single-cell level are made possible by these technology, enabling a deep understanding of the cellular composition and function within the tissue microenvironment [102]. They have been pivotal in studying complex diseases such as cancer, providing detailed insights into tumor heterogeneity, the immune microenvironment, and the spatial distribution of therapeutic targets.

#### Spatial transcriptomics: cataloging the cancer universe

**Fig. 3:**
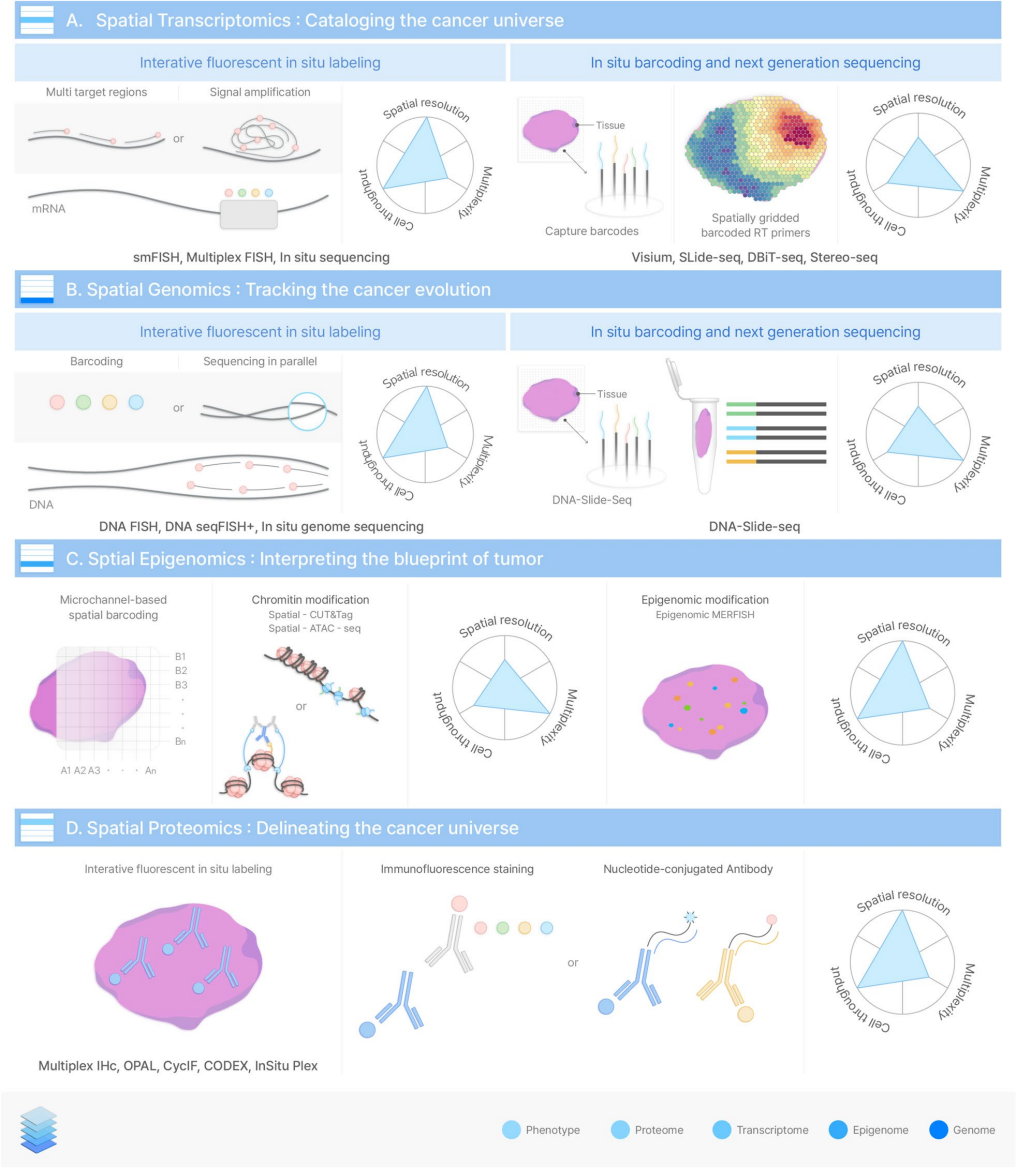
Technologies for mapping spatial landscape of cancer biology

While analyzing the specific regions of interest in pathology slides are important, it is also crucial to analyze the spatial context. Delineating the spatial context of cells and tissues is a fundamental biological issue in cancer research [103]. The spatial cellular context provides information on biological networks regarding how cells interact with their surroundings [104]. Technologies to analyze spatial biology have been developed to better observe the cellular context of tumor cells, just as telescopes have been developed for basic research on observing celestial bodies and their relationships [65, 105, 106] (Fig. 3). In particular, recent advances in spatial transcriptomics technologies have led us to observe a general pattern of molecular snapshots that provide biological information inferring cellular context. For this reason, spatial transcriptomics technologies were selected as ‘Method of the year 2020’ by Nature Methods [107].

Spatial transcriptomics were deciphered using two common approaches: microscopy and next-generation sequencing. The microscopic approach has the advantage of being able to visualize the expressed transcripts directly on the cells, and a sequencing-based approach enables unbiased deciphering of transcriptomes. Fluorescence *in situ* hybridization (FISH) is a historically method for imaging RNA transcripts using a fluorescence-tagged probe to detect target RNA molecules in cells [13, 15, 108–110]. Owing to the intrinsic limitation of spectral overlap of fluorescence channels, microscopic approaches have been developed to increase the multiplexity of target genes with higher detection accuracy. Initially, single-molecule FISH, which can detect RNA molecules within cells, used single probes for each transcript [111, 112]. Targeting different regions of the transcript with the same fluorescence has been developed to enhance the signal-to-noise ratio, enabling quantification of gene expression [113, 114]. An additional approach was developed to increase the signal intensity using a signal amplifier. RNAscope [115], for example, implements a Z-probe to hybridize at the target sequence and fluorescence labels to amplify the signals. These FISH methods have limitations in terms of the number of detectable markers due to the spectral overlap of fluorescence channels. Nonetheless, these methods provide high sensitivity and specificity with ease of use, allowing researchers to quantify their genes of interest with spatial location [116–118]. Therefore, FISH has been widely used to map precise spatial information of tumor-specific biomarkers in various tissue samples [119, 120].

Owing to the inherent limitations of the microscopic approach, various methods have been developed to increase the multiplexity of the target genes by iteration. In 2014, a sequential *in situ* hybridization method, seqFISH (commercialized by Spatial Genomics), was developed, which increased the number of detectable genes through iterative hybridization of fluorescent probes [121]. A series of fluorescent signals identified unique genes distinguishing up to thousands of genes. The original model had 24 encoding probes defined for each target in every hybridization round, making it possible to demonstrate up to 12 genes because of the expensive and time-consuming process. SeqFISH+, developed in 2019, utilizes a signal amplifier to enhance the signal detection ratio, increasing multiplexity by up to 10,000 genes in single cells [122]. Another spatial transcriptome localization method based on FISH, MERFISH(commercialized as Vizgen), a multiplexed error-robust FISH method, was published in 2015 [59]. It implements a probe with two flanking signal amplifier regions to efficiently increase the signal sensitivity while reducing the reaction time. The target probe, which has anchoring sites for the fluorescent probes, was designed for each gene. MERFISH increased specificity by adopting a coding scheme for error detection, which may arise during the molecular iteration step. An additional engineering approach with a highly increasing signal amplification step was used to increase the signal intensity to enhance the detection sensitivity and sensitivity of MERFISH [123]. Multiplex smFISH has been implemented to identify hundreds of genes, identify immune cells and cancer cells, and dissect the role of the TME in mesenchymal-like state transition [124]. Nanostring also developed CosMx, a spatial RNA and protein profiling method based on a single-cell imaging platform [60]. Microscopy-based iterative FISH imaging was used to read the expressed genes and proteins at a single-cell resolution. Multiplex smFISH, like SeqFISH and MERFISH, has greatly increased the number of spatially localized target genes, enabling the *in situ* characterization of cell states and cell types.

In addition to the quantification of target gene expression, *in situ* sequencing approaches have been developed that can directly sequence certain regions of transcripts in the tissue. An imaging-based microscopic approach requires sufficient amplification of the signals to be detected; therefore, it implements the amplification step of genetic molecules. The first *in situ* sequencing method using padlock probes was published in 2013 (commercialized as Xenium, 10X genomics) [58]. It can quantify known target genes by sequencing barcoded padlocks or detecting single-nucleotide variants in target regions using a gap-filling strategy. The padlock probes were hybridized to the target reverse-transcribed genes. After filling the gap, the phi29 enzyme, which amplifies the circle-shaped genetic molecules, processes the rolling circle amplification (RCA). The barcodes located in amplified products are then sequenced by sequence-by-ligation, modified to the sequence-by-hybridization method in the latter version, increasing the multiplexing capacity [125, 126]. Recent approaches to *in situ* sequencing are trying to proceed with sequencing without mRNA-to-cDNA conversion owing to the low efficiency of reverse Transcription [127]. ISS has been applied in breast cancer to analyze the cellular heterogeneity of tumor tissues [58, 128]. Fluorescence *in situ* sequencing (FISSEQ; commercialized as ReadCoor) is an untargeted *in situ* sequencing method that does not require predefined target probes [64]. Instead of using the target probe to amplify the sequence, FISSEQ amplifies every circularized cDNA in the cell matrix. The RCPs were sequenced by oligonucleotide ligation and detection (SOLiD) chemistry. FISSEQ has the advantage of being able to sequence RNA products in a cell matrix. Together with expansion microscopy, tumor microenvironments have been thoroughly deciphered by spatially mapping 297 tumor-related genes [129, 130]. STARmap, a spatially resolved transcript amplicon readout mapping, uses two target probes that can directly hybridize to RNA; and adopts hydrogel-tissue chemistry for high-resolution volumetric imaging [131].

Unlike other microscopic approaches that require the design of target probes, sequencing-based spatial transcriptome analysis enables an unbiased patterning of the transcriptome. ‘Spatial transcriptomics’ (commercialized as Visium, 10X genomics) is a recent method that enables RNA sequencing via *in situ* poly(A) tail capturing of tissue section [61]. Spatial information was retrieved through the spatial barcode present in the poly(dT) capture probes. Initially, the resolution of the spatial barcoding was 100 µm in diameter, limiting the cell resolution to ~30 cells. This technology is still being developed to improve spatial resolution and information depth. As it implements the spatial barcoding process, its spatial resolution is obtained from the density of the barcodes. Following the random barcoded bead array to physically lower the spatial resolution [62, 132], computational methods to infer lower resolutions by deep learning have been developed [133, 134]. Additionally, there was an intrinsic limitation that only the 3’ cDNA count could be recovered due to the poly(dT) barcoding; however, research to increase the information depth of the *in situ* capturing technology are actively being conducted, such as parallel sequencing with nanopore sequencer to obtain isoform sequence or inferred CNV analysis based on the gene counts [135–138]. *In situ* cer cohorts were stratified into clusters with distinct cellular compositions, suggesting that intrinsic subtype classification can be connected to clinical outcomes [139]. Heterogeneous subpopulations were identified in ductal carcinoma *in situ* of breast cancer and provided predictive biomarkers such as GATA3 dysfunction, PIK3CA mutations, and PgR negativity [41]. Additional genetic heterogeneity was dissected in cutaneous malignant melanoma in a spatial context to identify the factors regulating tumor progression and clinical outcome [140]. Moreover, tumor microenvironment characterization was thoroughly investigated by analyzing the spatial distribution of tumor architectures [141–145]. A single-cell tumor immune atlas has been suggested to stratify the immune microenvironment in tumor sections [146]. The interaction of FAP+ fibroblasts and SPP1+ macrophages has been observed in colorectal cancer, suggesting a possible tissue remodeling mechanism and potential intervention targets [48]. Novel biomarkers, such as the tumor boundary interfacial marker cilia gene or epithelial marker N-cadherin 2, were suggested by spatial molecular subtyping [147]. Additionally, spatially barcoded DNA nanoball-patterned arrays have been implemented for the *in situ* capture of transcriptomes, greatly increasing the spatial resolution [148]. Increasing research is being conducted using *in situ* capturing techniques to map the gene counts expressed in tissue slides, which will greatly help spatial cell mapping in clinical samples.

#### Spatial omics: towards onco-verse

Observing gene expression helps generalize the overall pattern of the tissue; however, it is not sufficient to fully understand cellular dynamics. Transcriptomics is an intermediate dimension representing genomic aberrations and inferring functional proteins regulated by epigenomics. By integrating multi-dimensional cellular information, it is possible to build a complete map for understanding cellular dynamics and interpreting the cell type, state, differentiation, and function. Recent progress in spatial-omics technologies has aided in the in-depth characterization of tumors in a spatial context.

#### Spatial genomics: tracking the cancer evolution

Spatial genomics is especially important in cancer biology because cancer progression is determined by the underlying genetic aberrations. As FISH detects gene expression by hybridizing the fluorescence probes to the target genes, it is used to identify genomic aberration [149, 150]. FISH-based *in situ* DNA analysis techniques can detect not only the spatial location, but also the chromosomal location within the nucleus [151, 152]. Advances in DNA FISH technologies have led to the identification of cell types using probes targeting single-nucleotide polymorphism [153]. Recently, sequential DNA FISH methods have been introduced to increase the multiplexity of the target DNA loci. DNA seqFISH+, an iterative FISH method, can target thousands of loci in cells, which can aid in analyzing the genomic organization. In addition, *in situ* genome sequencing (IGS) is a method for sequencing genomes using *in situ* imaging, preserving the spatial context and base-pair resolution sequencing [154]. It amplifies the genome in its native spatial context using TN5-assisted library preparation and rolling circle amplification *in situ*. After *in situ* imaging of the UMI, the recovered amplicons were sequenced using *ex situ* paired-end sequencing. By matching the molecular *in situ* UMI with the *ex situ* sequencing data, the DNA sequence can be mapped into the spatial context. Another barcoding approach was performed using Slide-seq [62], an *in situ* capturing transcriptomics method [155]. It uses a spatially barcoded bead array to capture spatially resolved genomic features and sequence DNA. Such spatial genome mapping technologies aid in characterizing tumor heterogeneity in tissue sections, comprising spatial clonal populations via genomic aberration.

#### Spatial epigenomics: interpreting the blueprint of tumor

Spatial epigenomics is rapidly evolving research field of analyzing the spatial organization of epigenetic modification which regulates the gene expression patterns. Epigenomics is an important regulatory tool that controls gene expression. Alterations in epigenetics are functionally important and increasing research has highlighted the clinical relevance of epigenetics in tumor studies. Growing epigenetic chemistry, such as Cut & Tag [156], ATACseq [157], ChIPseq [158]c, and Methyl-seq [40], has been developed to analyze histone modifications and chromatin accessibility. Recently, *in situ* spatial barcoding approaches (commercialized by AtlasXomics) have been introduced to add the spatial modality to epigenetic chemistry. Spatial-CUT&Tag [159] and Spatial-ATAC-seq [160] are microchannel-guided pixel-barcoding methods for profiling spatial histone modification and chromatin accessibility, respectively. It was built on the same chemistry as DBiT-seq [63], a microfluidic barcoding-based spatial omics technology. During each chemistry, a set of barcodes was delivered in two perpendicular rounds, resulting in a 2-dimensional barcoded grid of tissue pixels. Additionally, the same group that developed the MERFISH method has developed a method for spatially resolved epigenomic profiling of single cells, which utilizes *in situ* tagmentation and transcription followed by multiplexed imaging [161]. This techniques offer a higher subcellular resolution in constructing the spatial atlas of epigenomic enhancers. Spatial epigenome profiling technologies are in the early stages of development but are expected to offer a solution for mapping epigenetic regulation in tumor research [162].

#### Spatial proteomics: delineating the cancer landscape

Spatially resolved proteomic profiling has historically attracted the interest of researchers. H&E and immunofluorescence staining have been used to distinguish the cells for tumor characterization. The immunofluorescence staining technique itself has a limitation in the number of target proteins owing to the spectral overlap of the fluorescence probes. Spatially resolved protein profiling techniques have been developed to increase the number of proteins that co-localize.

Iterative fluorescence barcoding techniques are widely used to increase the number of target proteins. Similar to multiplex FISH methods, multiplex immunostaining methods have acquired multiplexity up to dozens of times by iteration of fluorophore tagging and stripping steps [163–165]. Owing to the harsh environment in which the tissue goes through during the iterative process, there is a limitation in multiplexity. To increase sensitivity, gentler methods to remove the fluorescence signal remaining from previous rounds were developed instead of stripping antibodies, such as fluorophore bleaching or intermediate reporter probes. For example, the co-detection by indexing method, CODEX (commercialized by Akoya Biosciences), is widely used as a tool to decipher spatially resolved proteins with up to 60 targets [166, 167]. This method uses a DNA-conjugated antibody to hybridize to target proteins and detect existing antibodies by indexing fluorescence-tagged nucleotides. This method guarantees a single-antibody staining procedure and simple indexing chemistry [47]. Its ability to profile highly multiplexed protein signatures enables the comprehensive characterization of the tumor microenvironment. It has been reported that spatial protein signatures have higher diagnostic accuracy for predicting immunotherapy response than genomic profiling approaches in anti-PD-1/ PD-L1 therapy [168]. Dozens of immune, tumor, and structural marker mapping was performed in FFPE fixed cutaneous T-cell lymphoma tissues to better study tumor immunology [169]. Other immune signatures are also spatially phenotyped in tumor microenvironments, such as immune cell infiltration patterns, neutrophil extracellular traps, intrafollicular memory CD4+ T-cells, etc. [170–172]. Spatial proteomics is a powerful discovery tool for analyzing cell biology at the functional level [173].

#### Spatial multi-omics: into the cancer multiverse

Compared to conventional approaches that target single-dimensional molecular targets for spatial profiling, multidimensional molecular information provides a better understanding of cellular mechanisms [174]. By integrating the’ omics’ profiles, researchers seek to interpret the systemic function of cancer biology. A growing number of research is reporting tools that integrate two or more dimensions of molecular targets. For instance, DBiT-seq suggested a microchannel-based spatial barcoding system, suggesting a solution for providing spatial modality in omics profiling technologies [63]. To co-profile mRNAs and proteins via next-generation sequencing while preserving the spatial context, they introduced antibody-derived DNA tags with poly-A tails to detect the target proteins and spatially barcoded the DNA tags and mRNAs prior to sequencing. To increase the number of target proteins, they recently integrated the CITE-seq, a high plex protein and whole transcriptome sequencing technology with the spatial barcoding system [175]. The group has also suggested a similar approach of endowing spatial modality via microfluidic barcoding to other omics assays, such as epigenetics [157, 159, 160].

Another approach integrates the existing spatial profiling to obtain molecular multi-dimension. SM-Omics is an automated spatial multi-omics profiling platform published in 2022 [176]. It integrates previously introduced methods, such as H&E staining, DNA-conjugated antibody-based protein measurements or immunofluorescence staining, with spatial transcriptomics techniques to profile the simultaneous analysis of spatially resolved RNAs and proteins [175]. This study demonstrated the combined profiling of RNA and protein expression in a mouse cancer model to characterize tissue niches with higher information depth. Increasing research has been conducted to provide spatial modality for two or more omics profiling technologies. Challenges still exist in combining multidimensional molecular information, but spatial multi-omics mapping offers a wide range of molecular information that aids researchers in defining cellular phenotypes, understanding cell-cell interactions, and identifying spatially expressed biomarkers in cancer. We envision that cancer biology should move towards spatial multi-omics profiling to systemically analyze the functional mechanism of the tumor.

Integrating spatial omics within the broader multiomics framework is pivotal for unraveling the complex biology of diseases. This approach is increasingly reshaping clinical trials and therapeutic strategies with its insightful revelations. For instance, the study by Zhang et al. (2023) serves as an example, where spatial transcriptomics and proteomics are employed to intricately map the tumor microenvironment in hepatocellular carcinoma [177]. Their work provides nuanced insights into the responses to neoadjuvant therapies, such as nivolumab and cabozantinib, enhancing our understanding of these treatments in virtual clinical trial settings. Complementing this, the research by Ruiz-Martinez et al. advances this integration by combining genomic, transcriptomic, and proteomic data within an agent-based model [178]. This model simulates the spatial dynamics of tumor growth and the effects of systemic immunotherapy, offering a holistic view of tumor-immune interactions. Furthermore, Song et al. demonstrate the application of artificial intelligence to analyze these multiomic datasets [179]. They focus particularly on histopathological images, which, when enriched with genomic and transcriptomic data, become powerful diagnostic tools in computational pathology. Collectively, these studies underscore the significance of multiomic data integration, where each omics layer contributes to a comprehensive spatial biological context. Such a granular understanding is instrumental in guiding precise and effective medical interventions.

#### The era of spatial (pathology) atlas will lead to next-generation diagnostics and therapeutics

With the decrease in the cost of next-generation sequencing, discovering the genomic, transcriptomic, and proteomic landscape, and exploring targets of interest have become possible with flourishing spatial technologies. Since the inauguration of the Human Genome Project in 1990, the Cancer Genome Anatomy Project (1997), Cancer Genome Atlas (2006), Human Cell Atlas (2016), and many other projects have sought to build a database or atlas of the landscape of human cancers. The next step is to build a spatial atlas or pathology atlas that comprehensively maps the genetic landscape of cells in heterogeneous tumor microenvironments and discover spatially relevant therapeutic markers.

The role of the spatial assay techniques varies according to the scalability of cell throughput and information depth of genetic molecules. Large-scale spatial omics technologies are capable of mapping the spatial pattern in tissue landscape, mostly focusing on discovering the spatial heterogeneity of tumors and the spatial composition of tumors (Table 1). Spatial landscaping technologies allows researchers to have the global view of tumor architecture, allowing for the identification of general trends and patterns in the molecular biology of the tumor. Conversely, targeted sampling technologies enable a detailed exploration of genetic information within selected subregions of the tissue. Such targeted sampling approaches focus on specific areas of interest, providing nuanced insights by integrating conventional chemistries for in-depth profiling. This method circumvents the need to survey entire slides, directing resources and attention to areas of greatest interest or variability within the sample. By homing in on these select subregions, Targeted sampling not only optimizes the depth and relevance of genetic information obtained but also enhances the efficiency of the study. This focused approach enables a more effective comparison across a large cohort of patients by analyzing representative samples, thus broadening the scope of data dimensionality and enabling a detailed examination of localized biological phenomena. Therefore, this approach is particularly suited for the discovery of novel diagnostic markers and therapeutic targets, rather than the broad patterning of spatial landscapes on a large scale (Table 2).

**Table 1:** Biological findings using spatial profiling technologies in cancer research

**Target**	**Technology**	**Findings Types**	**Biological Findings**	**Cancer Type**	**Reference**	**Stats ( gene/cells (gc) or gene/mm^2 (gm) )**
RNA	Visium	Biomaker	CNVs (Copy Number Variations), such as MYC and PTEN, occur early stage of cancer	Prostate cancer	Erickson et al. (2022) [138]	3500 genes / 7850µm^2 (100µm diameter spot)
	Visium	Biomarker	Upregulated cilia gene expression on tumor-normal cell interaction sites	Melanoma	Hunter et al. (2021) [147]	500-3000 genes / 1600µm^2 (55µm diameter spot)
	Visium	Biomarker	GATA3 mutation upregulates epithelial-to-mesenchymal transition and angiogenesis	Ductal Carcinoma In Situ (DCIS) of Breast cancer	Nagasawa et al. (2021) [41]	2928 genes / 1600µm^2 (55µm diameter spot)
	Visium	Biomarker	Macrophage population enhances inflammatory gene expression, including T-cell recruiting chemokine	Prostate Cancer	Tuong et al. (2021) [180]	
	Visium	Biomarker & Prognosis	CDH12-enriched tumors indicate poor clinical outcome, but superior response to ICT	Bladder cancer	Gouin III et al. (2021) [181]	>1250 UMIs / 1600µm^2 (55µm diameter spot)
	Visium	Heterogeneity	Heterogeneous response to 5ARI treatment	Prostate cancer	Joseph et al. (2021) [182]	
	Visium	Heterogeneity	Cell type deconvolution indicates T cell interaction	HER2-positive breast cancer	Andersson et al. (2021) [133]	0-200 cells / 7850µm^2 (100µm diameter spot)
	Visium	Heterogeneity	Heterogeneity with discoveries of novel cell states and unknown multicellular communities	Carcinoma	Luca et al. (2021) [183]	
	Visium	Heterogeneity	Spatial distribution of hypoxia-related heterogeneity	Pancreatic Ductal Adenocarcinoma (PDAC)	Sun et al. (2021) [141]	2178-2541 genes / 7850µm^2 (100µm diameter spot)
	Visium	Heterogeneity	Heterogeneous cell-type composition in each location	Pancreatic cancer	Ma et al. (2022) [184]	
	Visium	TME	Tumor-specific keratinocyte (TSK) cells serve as a hub for intercellular communication	Cutaneous Squamous Cell Carcinoma (cSSC)	Ji et al. (2020) [144]	~1200 genes / 9500µm^2 (110µm diameter spot)
	Visium	TME	Generated Single-cell Tumor Immune Atlas	13 types of cancer	Nieto et al. (2021) [146]	
	Visium	TME	Tumor growth when arginase-1 expression by myeloid cells	Neuroblastoma	Van de Velde et al. (2021) [185]	
	Visium	TME	Atlas of human breast cancer; Immune related composition within tumor	Breast cancer	Wu et al. (2021) [139]	/ 1600µm^2 (55µm diameter spot)
	Visium	TME	Metastatic microenvironment contains immunosuppressive cells which have better metabolic activity.	Colorectal cancer	Wu et al. (2022) [27]	1-10 cells per spot
	Visium	TME	Interleukin-10-releasing myeloid cells causes immunosuppressive TME by driving T cell exhaustion	Glioblastoma	Ravi et al. (2022) [145]	4-22 cells per spot
	Visium	TME	High FAP and SPP1 leads to therapeutic failure	Colorectal cancer	Qi et al. (2022) [48]	2051-4937 genes per spot
	Visium	TME	Tgfbr2 knockout converted TME leading to fibroblast activation	Lung cancer	Dhainaut et al. (2022) [186]	
	Visium	TME & Heterogeneity	Complex heterogeneous gene expression of lymphoid area close to tumor	Melanoma (Stage III Cutaneous Malignant)	Thrane etal. (2018) [140]	
	Visium	TME & Heterogeneity	Detection of tumor subclones of each ductal region and T cell adjacent to the tumor	Ductal Carcinoma In Situ (DCIS) of Breast cancer	Wei et al. (2022) [187]	19-1562 genes / 1600µm^2 (55µm diameter spot)
	Visium	TME & Heterogeneity & Biomarker	Cell-to-cell interaction exists spatially, creating restricted enriched clusters	Pancreatic Ductal Adenocarcinoma (PDAC)	Moncada et al. (2020) [142]	1000 genes / 7850 µm^2 (100µm diameter spot)
	MERFISH	TME	Cancer cells and immune cells interaction leads to mesenchymal-like states	Glioblastoma	Hara et al. (2021) [124]	135 genes / 14181 cells
	MERFISH	TME	Heterogeneous niches having different response to immune checkpoint blockade	Hepatocellular carcinoma	Magen et al. (2022) [188]	
	ISS	Biomarker	Observation of gene mutations and profiling gene expression	Breast cancer	Ke et al. (2013) [58]	256 genes / 1-35 cells
	ISS	Biomarker	Uncovering sources of pro-angiogenic signaling, role of mesenchymal-like cancer cells	Glioblastoma	Ruiz-Moreno et al. (2022) [189]	1.1 million cells
	ISS	Heterogeneity	Detection of intratumoral heterogeneity with its specific gene expression patterns	Breast cancer	Svedlund et al. (2019) [128]	91 genes
	Fisseq	Biomarker	ExSeq enabled better detection of gene expression	Breast cancer	Alon et al. (2021) [190]	297 genes / 2395 cells
	RNAscope	Biomarker	Validation of Accurate gene expression detection	Gastric cancer	Tamma et al. (2018) [191]	
	RNAscope	Heterogeneity	Heterogeneous spatial distribution of HER2 and ER gene expression	Breast cancer	Annaratone et al. (2017) [119]	38191 cells
	RNAscope	Heterogeneity	TERT gene expression spatially heterogeneous	10 human cancer cell lines	Rowland et al. (2019) [120]	3 copies of genes / 55-204 cells
Protein	Nanostring	Biomarker	MEK inhibitor and JAK/STAT3 pathway inhibitor can be a potential solution for tumorigenesis	Medulloblastoma	Zagozewski et al. (2022) [192]	56 proteins / 12 ROIs
	Nanostring	Biomarker & Heterogeneity	Immune checkpoint protein supporting heterogeneity	Metastatic prostate cancer	Brady et al. (2021) [193]	100-900 genes, 8-35 proteins / 1200 cells per ROI [168 ROI (500µm size ) ]
	Nanostring	Prognosis & biomarker	Observation of gene expression in tumor due to adjuvant chemotherapy can further be used for prognosis	Triple Negative Breast Cancer (TNBC)	Kulasinghe et al. (2022) [194]	68 targets /
	Nanostring	TME	Discovered fetal-like reprogramming of TME causing Immunosuppressive onco-fetal ecosystem	Hepatocellular Carcinoma	Sharma et al. (2020) [195]	96 genes / 212000 cells [12 ROI (500µm size ) per slide]
	Nanostring	TME	Multicellular interaction networks that underlie immunologic and tumorigenic processes	Colorectal cancer	Pelka et al. (2021) [196]	204 genes / 371223 cells [45 circular regions of interest measuring 500 μm in diameter]
	Nanostring	TME	Anti-tumor immunity failure due to increased immune suppression within TDLN (Tumor Draining Lymph Node)	Melanoma	Van Krimpen et al. (2022) [197]	730 genes, 58 protein markers / 5 ROIs per patient
	Nanostring	TME	Bacterial burden was significantly high in lung tumor, corresponding to oncogenic pathways	Lung cancer	Wong-Rolle et al. (2022) [198]	
	Nanostring	TME & Biomarker	Mechanism of Myofibroblast avoiding the adaptive immune resopnse	Pancreatic Ductal Adenocarcinoma (PDAC)	Han et al. (2022) [199]	78 genes, 21 proteins / 24 ROI [24 ROI (300µm size ) ]
	Nanostring	TME & Biomarker	Gene expression difference between Primary and Lymph node metastasis from oropharyngeal SCC (OPSCC)	Head and Neck Squamous cell Carcinoma	Sadeghirad et al. (2022) [200]	
	Nanostring	TME & Heterogeneity	Nerves adjacent to tumor exhibits high stress and growth response	Oral cancer	Schmitd et al. (2022) [201]	8162 genes / All ROI (unknown diameter)
	Nanostring	TME & Prognosis	Proteomic changes were detected, and can be used for prognosis for neo-adjuvent HER2-targeted therapy	HER2-positive Breast Cancer	McNamara et al. (2021) [202]	40 biomarkers / 122 samples with 2 ROIs each
	mIHC	TME & Biomarker	Different response to CSF1R blockade from two distinct TAM(Tumor-associated Macrophage)	Colon Cancer	Zhang et al. (2020) [203]	
	mIHC	TME	Heterogenous TME (Tumor Microenvironment) has different response to PC (preoperative chemotherapy)	Colorectal Cancer	Che et al. (2021) [204]	
	mIHC	TME	TAM (Tumor-associated Macrophage) derived from different types of myeloid cells causes heterogeneity	Glioblastoma	Pombo Antunes et al. (2021) [205]	
	mIHC	TME& Prognosis	PDAC Tumor immune microenvironment reflected a low immunogenic ecosystem and correlates with patient survival.	Pancreatic Ductal Adenocarcinoma (PDAC)	Mi, Haoyang, et al. (2022) [206]	27 markers
	mIHC	TME& Prognosis	Leukocyte heterogeneity in PDAC TiME affects patient survival	PDAC	Liudahl, Shannon M., et al. (2021) [207]	27 markers
	mIHC	Prognosis	Neoadjuvant chemotherapy response prediction using H&E and mIHC Tissue Microarray data in muscle-invasive bladder cancer (MIBC)	Bladder Cancer	Mi, Haoyang, et al. (2021) [208]	
	mIHC	Prognosis	Patient survival prediction model using mIHC slides (CD8, CD20, k56) in ovarian cancer	Ovarian Cancer	Nakhli, Ramin, et al.(2023) [209]	3 markers
	CODEX	TME	Identification of distinct cellular neighborhoods and their impact on both TME and survival rate	Colorectal Cancer	Schürch et al. (2020) [210]	56 markers
	CODEX	TME & Biomarker	Low expression of intrafollicular CD4 expression indicates early failure	Follicular lymphoma	Mondello et al. (2021) [172]	23 markers
	CODEX	TME & Biomarker	Discovered spatial biomarker, SpatialScore, which causes pembrolizumab response	Cutaneous T cell lymphomas (CTCL)	Phillips et al. (2021) [211]	56 markers / 117170 cells
	CODEX	Biomarker & Prognosis	CDH12-enriched tumors indicate poor clinical outcome, but superior response to ICT	Bladder cancer	Gouin et al. (2021) [181]	35 markers

**Table 2:** Biological findings by targeted sampling-based sorting technologies in cancer research

**Target**	**Technology**	**Finding Type**	**Biological Findings**	**Cancer**	**Reference**	**Stats ( gene/cells (gc) or gene/mm^2 (gm) )**
RNA	LCM	Biomarker	AVR7 is a tumor suppressive gene in gastric resistant prostate cancer	Prostate cancer	Cato et al.(2019) [212]	
	LCM	Biomarker	COL1A1 is a promising therapeutic marker in glioma	NSCLC	Baldelli et al(2022) [213]	6-60 cells
	LCM	Biomarker	Invasive lobular breast cancer's stroma and CAF pathway discovery and two genes were influenced survival rates.	Invasive lobular breast cancer	Gómez-Cuadrado et al.(2022) [214]	
	LCM	Biomarker	Detection of EGFR and KRAS mutation with few cells, approximately 50 tumor cells	Lung adenocarcinoma	Chowdhuri et al. (2012) [215]	As few as 50 cells
	LCM	Biomarker	Efficiency of detecting EGFR and KRAS gene mutations increased significantly with LCM	Lung cancer	Malapelle et al. (2011) [216]	
	LCM	Heterogeneity	Recurrent nasopharyngeal carcinoma has a differential gene expression from non-recurrent tumor.	Nasopharyngeal cancer	Tay et al.(2022) [217]	
	SLACS	Biomarker	A-to-I editing events in a specific gene has a correlation with the therapeutic response	Triple Negative Breast Cancer	Lee et al. (2022) [55, 95]	3-5 c
DNA	LCM	Biomarker	LOH of 10q23.3 marker for metastatic progression	Node-positive prostate cancer	Rubin et al. (2000) [218]	
	LCM	Biomarker	Allelic loss at chromosome p16 and p53 consistent during cancer progression	Metastatic bladder cancer	Cheng et al. (2001) [219]	
	LCM	Biomarker	LOH (Loss of Heterozygosity) detection in tumor	Inflammatory Breast Cancer	Bertheau et al. (2001) [220]	500 cells / 5000 cells
	LCM	Biomarker	Allelic loss of activated X chromosome related to carcinogenesis and progression	Bladder cancer	Cheng et al. (2004) [221]	400-600 cells
	LCM	Biomarker	Observed AMACR (Alpha-methylacyl-coenzyme A racemase) regulation	Colon Adenoma-carcinoma	Zhang et al. (2009) [222]	
	LCM	Biomarker	Detection of somatic mutations	Various solid tissues and lobular carcinoma	Ellis et al. (2020) [74]	100-1000 cells
	LCM	CTC genotyping	CTC genotyping in glioma	glioma	Zhu et al.(2022) [223]	
	LCM	Heterogeneity	Heterogeneity based on the observations of LOH (Loss of Heterozygosity)	Breast cancer	Wild et al. (2000) [224]	
	LCM	Heterogeneity	Detection of nonrandom X chromosome inactivation in different regions of same tumor sample	Bladder Carcinoma	Cheng et al. (2002) [225]	400-600 cells
	LCM	Heterogeneity	Detection of geneitc divergence during clonal evolution	Cell renal cell carcinoma	Jones et al. (2005) [226]	400-1000 cells
	LCM	Heterogeneity	Identification of frequent genetic divergence during metastases	Cutaneous melanoma	Katona et al. (2007) [227]	400-1000 cells
	LCM	Heterogeneity	Structural variant analysis with LCM+ grouping tumor types	Post-pubertal testicular germ-cell tumours	Bryce et al.(2019) [73]	
	LCM	Heterogeneity	Somatic mutation pattern analysis of cancers	Various types	Olafsson et al.(2021) [228]	few hundreds cells
	SLACS	Heterogeneity	Genomic landscape of the cells in 3D tumor mass	Breast cancer	Kim et al. (2018) [92]	3-5 cells
	SLACS	Prognosis	Mapping of clonal changes within hematopoietic lineages to performing prognosis liquid cancer	Myeloma and leukemia	Jeong et al. (2023) [96]	
Protein	LCM	Biomarker	Using RRPA and LCM found cellular signaling protein in breast cancer	Breast cancer	Cowherd et al.(2004) [229]	
	LCM	Biomarker	Observation of 12 novel TVM (tumor vascular markers)	Ovarian cancer	Buckanovich et al. (2007) [230]	-2000 cells
	LCM	Biomarker	Finding a connection with protein and subcellular structure names invadosome about cancer	Cancer specimen	Ezzoukhry et al.(2018) [83]	312 proteins enriched / 100, 350, 3000, 10000, 40000 cells
	LCM	Biomarker	NMNT is a marker which affects CAF(Cancer-associated Fibroblast).	glioblastoma	Lam et al.(2022) [87]	40,000,000 µm^2 for proteomics
Epigenome	LCM	Biomarker	Detected 766 up or down-regulated genes with subtype comparisons	Lung adenocarcinoma	Selamat et al. (2012) [231]	766 genes
	LCM	Heterogeneity	Using RRBS with LCM and found DNA methylation pattern of adrenoncoroticla	adrenocortical carcinoma	Schillebeeckx et al.(2013) [232]	
	LCM	CTC methylation profiling	Epigenetic features of CTC with LCM	Lung cancer	Zhao et al.(2021) [86]	10, 50 cells
DNA + RNA	LCM	Biomarker	Finding a biomarker for TNBC patients with phase2 neoadjuvant therapy	Breast cancer	Jovanovic et al. (2017) [233]	
	LCM	Heteogeneity	CNV and gene expression profiling in ROI of oral squamous cell carcinoma	Oral squamous cell carcinoma	Chen et al. (2022) [234]	230 cells
	LCM	Heterogeneity	Landcape of genomic and transcriptomic of Lung Adenomatous Premalignancy	Lung cancer	Krysan et al. (2019) [235]	
	LCM	Tumor subtyping & heterogeneity	regrouping cancer subtypes with proteome analysis which leads to overcome therapy resistance and targeting heterogeneity	TNBC	Zhu et al. (2021) [88]	50-200 cells
Protein + RNA	LCM	Biomarker	Identification cancer promoting stromal component in proteomic and transciptomic aspects in canine mammary tumors	canine mammary tumors	Poschel et al (2021) [236]	

**Fig. 4:**
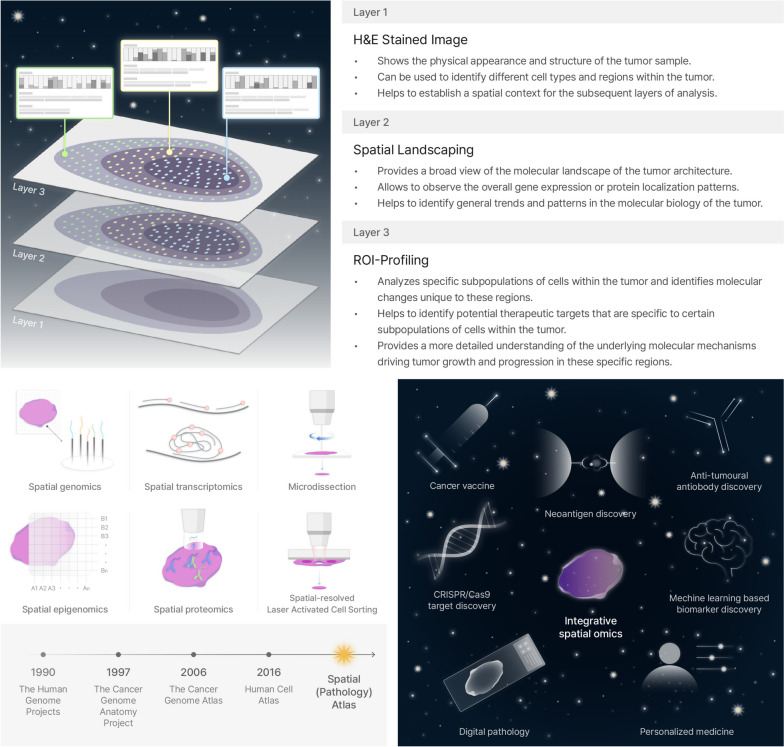
The era of spatial atlas will lead to next generation diagnostics and therapeutics

While recent advancements in multiplexed imaging technologies have expanded our capacity to obtain spatial cellular information, it is important to acknowledge the enduring significance of Hematoxylin and Eosin (H&E) staining in routine laboratory and clinical practice (Fig. 4). H&E staining remains the cornerstone of histopathological analysis, serving as the foundation upon which tissue architecture and pathological states are primarily assessed. Despite its limitations in molecular specificity, H&E's ability to delineate basic cellular structures has proven invaluable, particularly in the realm of digital pathology. The integration of digital pathology with machine learning algorithms has unlocked new potentials for H&E-stained slides, which are abundant and rich in histological detail [237]. This synergy is vital in translating routine histological images into predictive biomarkers and prognostic tools. Studies leveraging computational techniques have demonstrated the efficacy of extracting clinically relevant information from H&E images, underscoring their utility in patient outcome correlations.

It is essential to acknowledge the synergistic potential between H&E staining and advanced spatial profiling technologies. The simplicity of H&E staining, with its ability to provide a fundamental overview of tissue architecture, is an asset. When H&E images are layered with the rich, molecular data from spatial profiling technologies, the resulting composite offers a more nuanced and comprehensive analysis than either method could provide alone. This integration allows for the extraction of an even greater wealth of information, leveraging the straightforwardness of H&E to contextualize and enhance the complex data obtained from spatial profiling. Therefore, as we navigate the trend towards more intricate spatial omics, the indispensable role of H&E staining must be highlighted, not only for its current applications in oncological studies but also for its potential to be combined with spatial profiling for superior analytical depth and insight into cancer research.

Spatial omics technologies has already begun to utilize spatial omics to discover diagnostic biomarkers combined with machine learning and digital pathology [238–240]. The field of spatial omics especially will be affected with machine learning-based or deep learning-based digital pathology, as image-based digital pathology provides feature or ROI extractions that have been impossible to extract with human experience. Therefore, artificial intelligence (AI)-based pathological feature extraction provides an attractive method to select ROIs for revealing next generation molecular diagnostic marker that distinguishes the pathological feature from other regions. In addition, discovering transcript sequences that are being discovered in cancers [95] will provide useful guidelines for designing mRNA-based cancer vaccines, neoantigen targeting chimeric antigen receptor T cell (CAR-T cell) therapy, gene editing therapeutics including CRISPR/Cas9, RNA interference, and many other therapeutics. In addition, profiling immune cells residing or infiltrating the cancers will be useful in anti-tumor antibody discovery.

The power of spatial omics technologies exponentially increases when combined with each other and when combined with non-spatial technologies, such as FACS, single-cell technologies, and other spatial assays, such as staining technologies or digital pathology. Understanding the advantages and disadvantages of different spatial technologies, provides opportunities to design combinations. For example, after performing seqFISH on a tissue section, the seqFISH data can guide targets of interest that can be isolated with SLACS to add a different data modality. A more complex example is the combination of single-cell technologies with CosMx technology to discover specific cell types with specific transcripts. Specific cells can then be labeled by in situ sequencing and isolated by LCM or SLACS to analyze their genome, proteome, or metabolome. The combinations are limitless; therefore, it is important to design an appropriate combination according to the biological question. This integration strategy fits well with the nature of cancer, which is extremely heterogeneous and complex to understand.

## Conclusion

Here, we have reviewed the current methods to investigate spatially relevant biological findings in tumor tissue. The study of the spatial context in oncology is extremely important in terms of addressing tumor heterogeneity, the tumor microenvironment, and novel spatial biomarkers to identify the mechanism underlying tumors. Technologies developed, to date, have mostly focused on addressing spatial transcriptomics; however, increasing research has reported the integration of two or more omics profiles to interpret the systemic function of oncology. Additionally, it is possible to proceed with in-depth analysis of significant regions in spatial context by combining the large-scale spatial omics profiling technologies and targeted sampling -based spatial omics technologies. In conclusion, the exploration of spatially relevant biological findings in tumor tissue is of utmost importance for unraveling tumor heterogeneity, the tumor microenvironment, and identifying novel spatial biomarkers that underlie the mechanisms of tumors. The potential for spatial omics continues to grow, and in the near future, we envision that next-generation diagnostics or therapeutics will bloom from these targets discovered using spatial omics technologies.

Tumors are complex structures composed of multiple cell types that interact with each other and their microenvironment, leading to the underlying cellular mechanisms driving tumor growth and progression. Spatial analysis in tumor research can provide important information regarding proximity, cellular composition, morphology, and structure. Significant technologies have been developed in cancer biology to investigate the overall tumor landscape or analyze the in-depth characterization of tumor regions of interest.

Schematic representative of the technologies available for spatial omics profiling in regions of interest (ROIs). LCM-IR uses the Infrared-activated polymer to glue out the regions of interest. LCM-UV dissects out the regions or interest by using an ultraviolet laser. SLACS punches out the regions of interest in target-per-second speed by IR-pulse laser-activated vaporization. After the isolation of the regions of interest, targets can be applied to conventional molecular profiling assays such as DNA-sequencing, RNA-sequencing, and Mass-spec. DSP and IMC technologies employ special probes designed to target specific genes or proteins. These probes can be retrieved by illuminating or exciting particular regions of interest within a sample, profiling the associated molecular expressions.

Schematic representative of the technologies for mapping the spatial landscape of cancer biology in terms of profiling transcriptomics, genomics, epigenomics, and proteomics. Spatial transcriptomics and genomics: Most of the currently available spatial transcriptomics can be categorized into iterative fluorescent labeling methods and in situ barcoding methods. Spatial epigenomics: Technologies for spatial epigenomics are in its early stage, mostly focused on a microfluidic channel based on in situ spatial barcoding technology combined with conventional epigenomic profiling assays. Spatial Proteomics: Fluorescence-tagged antibodies are applied to spatially profile the spatial presence of the target protein.

The next step of human cell atlas would be building a pathology atlas that delineates the molecular landscape in heterogeneous tumor microenvironments. Furthermore, the applicability of spatial omics technologies is not limited to spatial atlasing, eventually leading to the next generation diagnostics and therapeutics.

## Data Availability

Not applicable.

## References

[1] Faguet GB (2015). A brief history of cancer: Age-old milestones underlying our current knowledge database. Int J Cancer.

[2] Hooke R (2003). Micrographia: Or Some Physiological Descriptions of Minute Bodies Made by Magnifying Glasses, with Observations and Inquiries Thereupon (Courier).

[3] J, M. (1838). Uber den feineran Bau and die For-man der Krankhauten Geschwulste. Berlin G Reimer.

[4] Hajdu SI (2004). The First Tumor Pathologist. Ann Clin Lab Sci..

[5] King D Friday, King LAC (1986). A Brief Historical Note on Staining by Hematoxylin and Eosin. Am J Dermatopathol..

[6] Cook HC (1997). Tinctorial methods in histology. J Clin Pathol.

[7] Yuan, Y. (2016). Spatial Heterogeneity in the Tumor Microenvironment. Cold Spring Harb. Perspect. Med. 6. 10.1101/CSHPERSPECT.A026583. 10.1101/cshperspect.a026583 PMC4968167 27481837

[8] Coons, A.H., Creech, H.J., and Jones, R.N. (1941). Immunological Properties of an Antibody Containing a Fluorescent Group.∗: 47, 200–202. 10.3181/00379727-47-13084P.

[9] Le Bouvier GL (1971). The Heterogeneity of Australia Antigen. Source J. Infect. Dis..

[10] Gall JG, Lou M, Kline P, Giles NH (1969). Formation and detection of RNA-DNA hybrid molecules in cytological preparations. Proc Natl Acad Sci U S A..

[11] Levsky JM, Singer RH (2003). Fluorescence in situ hybridization: past, present and future. J Cell Sci..

[12] Langer-Safer PR, Levine M, Ward DC (1982). Immunological method for mapping genes on Drosophila polytene chromosomes. Proc Natl Acad Sci..

[13] Huber D, Voith von Voithenberg L, Kaigala GV (2018). Fluorescence in situ hybridization (FISH): History, limitations and what to expect from micro-scale FISH?. Micro Nano Eng..

[14] Liegl B, Kepten I, Le C, Zhu M, Demetri GD, Heinrich MC, Fletcher CDM, Corless CL, Fletcher JA (2008). Heterogeneity of kinase inhibitor resistance mechanisms in GIST. J Pathol..

[15] Hu L, Ru K, Zhang L, Huang Y, Zhu X, Liu H, Zetterberg A, Cheng T, Miao W (2014). Fluorescence in situ hybridization (FISH): An increasingly demanded tool for biomarker research and personalized medicine. Biomark Res..

[16] Sanger F, Nicklen S, Coulson AR (1977). DNA sequencing with chain-terminating inhibitors. Proc Natl Acad Sci U S A..

[17] Lander ES, Linton LM, Birren B, Nusbaum C, Zody MC, Baldwin J, Devon K, Dewar K, Doyle M, Fitzhugh W (2001). Initial sequencing and analysis of the human genome. Nat..

[18] Hood L, Rowen L (2013). The human genome project: Big science transforms biology and medicine. Genome Med..

[19] The Cancer Genome Atlas https://www.cancer.gov/tcga.

[20] Herzenberg LA, Sweet RG, Herzenberg LA (1976). Fluorescence-activated cell sorting. Sci. Am..

[21] Macosko EZ, Basu A, Satija R, Nemesh J, Shekhar K, Goldman M, Tirosh I, Bialas AR, Kamitaki N, Martersteck EM (2015). Highly parallel genome-wide expression profiling of individual cells using nanoliter droplets. Cell.

[22] Tang F, Barbacioru C, Wang Y, Nordman E, Lee C, Xu N, Wang X, Bodeau J, Tuch BB, Siddiqui A (2009). mRNA-Seq whole-transcriptome analysis of a single cell. Nat Methods.

[23] Patel AP, Tirosh I, Trombetta JJ, Shalek AK, Gillespie SM, Wakimoto H, Cahill DP, Nahed BV, Curry WT, Martuza RL (2014). Single-cell RNA-seq highlights intratumoral heterogeneity in primary glioblastoma. Science (80-. ).

[24] Han S, Fu D, Tushoski GW, Meng L, Herremans KM, Riner AN, Geoge TJ, Huo Z, Hughes SJ (2022). Single-cell profiling of microenvironment components by spatial localization in pancreatic ductal adenocarcinoma. Theranostics.

[25] Navin N, Kendall J, Troge J, Andrews P, Rodgers L, McIndoo J, Cook K, Stepansky A, Levy D, Esposito D (2011). Tumour evolution inferred by single-cell sequencing. Nat..

[26] Almagro J, Messal HA, Elosegui-Artola A, van Rheenen J, Behrens A (2022). Tissue architecture in tumor initiation and progression. Trends Cancer.

[27] Wu Y, Yang S, Ma J, Chen Z, Song G, Rao D, Cheng Y, Huang S, Liu Y, Jiang S (2022). Spatiotemporal Immune Landscape of Colorectal Cancer Liver Metastasis at Single-Cell Level. Cancer Discov..

[28] Andersson A, Larsson L, Stenbeck L, Salmén F, Ehinger A, Wu SZ, Al-Eryani G, Roden D, Swarbrick A, Borg Å (2021). Spatial deconvolution of HER2-positive breast cancer delineates tumor-associated cell type interactions. Nat. Commun..

[29] Elaldi R, Hemon P, Petti L, Cosson E, Desrues B, Sudaka A, Poissonnet G, Van Obberghen-Schilling E, Pers JO, Braud VM (2021). High Dimensional Imaging Mass Cytometry Panel to Visualize the Tumor Immune Microenvironment Contexture. Front Immunol..

[30] Wortman JC, He TF, Solomon S, Zhang RZ, Rosario A, Wang R, Tu TY, Schmolze D, Yuan Y, Yost SE (2021). Spatial distribution of B cells and lymphocyte clusters as a predictor of triple-negative breast cancer outcome. npj Breast Cancer.

[31] Ma RY, Black A, Qian BZ (2022). Macrophage diversity in cancer revisited in the era of single-cell omics. Trends Immunol..

[32] Sautès-Fridman C, Petitprez F, Calderaro J, Fridman WH (2019). Tertiary lymphoid structures in the era of cancer immunotherapy. Nat Rev Cancer.

[33] Dagogo-Jack I, Shaw AT (2017). Tumour heterogeneity and resistance to cancer therapies. Nat. Rev. Clin. Oncol..

[34] Kulasinghe A, Monkman J, Shah ET, Matigian N, Adams MN, O’Byrne K (2021). Spatial Profiling Identifies Prognostic Features of Response to Adjuvant Therapy in Triple Negative Breast Cancer (TNBC). Front Oncol..

[35] Schmelz K, Toedling J, Huska M, Cwikla MC, Kruetzfeldt LM, Proba J, Ambros PF, Ambros IM, Boral S, Lodrini M (2021). Spatial and temporal intratumour heterogeneity has potential consequences for single biopsy-based neuroblastoma treatment decisions. Nat Commun..

[36] Ma L, Hernandez MO, Zhao Y, Mehta M, Tran B, Kelly M, Rae Z, Hernandez JM, Davis JL, Martin SP (2019). Tumor Cell Biodiversity Drives Microenvironmental Reprogramming in Liver Cancer. Cancer Cell.

[37] Janiszewska M, Liu L, Almendro V, Kuang Y, Paweletz C, Sakr RA, Weigelt B, Hanker AB, Chandarlapaty S, King TA (2015). In situ single-cell analysis identifies heterogeneity for PIK3CA mutation and HER2 amplification in HER2-positive breast cancer. Nat Genet..

[38] Gambardella G, Viscido G, Tumaini B, Isacchi A, Bosotti R, di Bernardo D (2022). A single-cell analysis of breast cancer cell lines to study tumour heterogeneity and drug response. Nat Commun..

[39] Lomakin A, Svedlund J, Strell C, Gataric M, Shmatko A, Rukhovich G, Park JS, Ju YS, Dentro S, Kleshchevnikov V (2022). Spatial genomics maps the structure, nature and evolution of cancer clones. Nat..

[40] Liu H, Zhou J, Tian W, Luo C, Bartlett A, Aldridge A, Lucero J, Osteen JK, Nery JR, Chen H (2021). DNA methylation atlas of the mouse brain at single-cell resolution. Nat..

[41] Nagasawa S, Kuze Y, Maeda I, Kojima Y, Motoyoshi A, Onishi T, Iwatani T, Yokoe T, Koike J, Chosokabe M (2021). Genomic profiling reveals heterogeneous populations of ductal carcinoma in situ of the breast. Commun Biol..

[42] Lin J-R, Wang S, Coy S, Lau KS, Santagata S, Sorger Correspondence PK, Chen Y-A, Yapp C, Tyler M, Nariya MK (2023). Multiplexed 3D atlas of state transitions and immune interaction in colorectal cancer. Cell.

[43] Dam, S. van, Baars, M.J.D., and Vercoulen, Y. (2022). Multiplex Tissue Imaging: Spatial Revelations in the Tumor Microenvironment. Cancers (Basel). 14. 10.3390/CANCERS14133170. 10.3390/cancers14133170 PMC9264815 35804939

[44] Fu T, Dai LJ, Wu SY, Xiao Y, Ma D, Jiang YZ, Shao ZM (2021). Spatial architecture of the immune microenvironment orchestrates tumor immunity and therapeutic response. J Hematol Oncol..

[45] Merkher Y, Weihs D (2017). Proximity of Metastatic Cells Enhances Their Mechanobiological Invasiveness. Ann Biomed Eng..

[46] Qi J, Sun H, Zhang Y, Wang Z, Xun Z, Li Z, Ding X, Bao R, Hong L, Jia W (2022). Single-cell and spatial analysis reveal interaction of FAP+ fibroblasts and SPP1+ macrophages in colorectal cancer. Nat Commun..

[47] Hickey JW, Neumann EK, Radtke AJ, Camarillo JM, Beuschel RT, Albanese A, McDonough E, Hatler J, Wiblin AE, Fisher J (2021). Spatial mapping of protein composition and tissue organization: a primer for multiplexed antibody-based imaging. Nat Methods.

[48] Qi J, Sun H, Zhang Y, Wang Z, Xun Z, Li Z, Ding X, Bao R, Hong L, Jia W (2022). Single-cell and spatial analysis reveal interaction of FAP+ fibroblasts and SPP1+ macrophages in colorectal cancer. Nat Commun.

[49] Wu, P.H., Gilkes, D.M., Phillip, J.M., Narkar, A., Cheng, T.W.T., Marchand, J., Lee, M.H., Li, R., and Wirtz, D. (2020). Single-cell morphology encodes metastatic potential. Sci. Adv. 6. 10.1126/SCIADV.AAW6938/SUPPL_FILE/AAW6938_TABLES_S1_TO_S5.XLSX. 10.1126/sciadv.aaw6938 PMC6976289 32010778

[50] Alizadeh, E., Castle, J., Quirk, A., Taylor, C.D.L., Xu, W., and Prasad, A. (2020). Cellular morphological features are predictive markers of cancer cell state. Comput. Biol. Med. 126. 10.1016/J.COMPBIOMED.2020.104044. 10.1016/j.compbiomed.2020.104044 33049477

[51] Noble R, Burri D, Le Sueur C, Lemant J, Viossat Y, Kather JN, Beerenwinkel N (2021). Spatial structure governs the mode of tumour evolution. Nat. Ecol. Evol..

[52] Almagro J, Messal HA, Elosegui-Artola A, van Rheenen J, Behrens A (2022). Tissue architecture in tumor initiation and progression. Trends Cancer.

[53] Marx, V. Method of the Year: spatially resolved transcriptomics 10.1038/s41592-020-01033-y. 10.1038/s41592-020-01033-y 33408395

[54] Emmert-Buck MR, Bonner RF, Smith PD, Chuaqui RF, Zhuang Z, Goldstein SR, Weiss RA, Liotta LA (1996). Laser capture microdissection. Science.

[55] Lee AC (2023). Epitranscriptomics of cancer microniches. Nat. Rev. Cancer.

[56] Liotta LA, Pappalardo PA, Carpino A, Haymond A, Howard M, Espina V, Wulfkuhle J, Petricoin E (2021). Laser Capture Proteomics: spatial tissue molecular profiling from the bench to personalized medicine. Expert Rev. Proteomics.

[57] Merritt CR, Ong GT, Church SE, Barker K, Danaher P, Geiss G, Hoang M, Jung J, Liang Y, McKay-Fleisch J (2020). Multiplex digital spatial profiling of proteins and RNA in fixed tissue. Nat Biotechnol..

[58] Ke R, Mignardi M, Pacureanu A, Svedlund J, Botling J, Wählby C, Nilsson M (2013). In situ sequencing for RNA analysis in preserved tissue and cells. Nat Methods.

[59] Chen KH, Boettiger AN, Moffitt JR, Wang S, Zhuang X (2015). Spatially resolved, highly multiplexed RNA profiling in single cells. Science (80-. ).

[60] He S, Bhatt R, Brown C, Brown EA, Buhr DL, Chantranuvatana K, Danaher P, Dunaway D, Garrison RG, Geiss G (2022). High-plex Multiomic Analysis in FFPE at Subcellular Level by Spatial Molecular Imaging. bioRxiv.

[61] Ståhl PL, Salmén F, Vickovic S, Lundmark A, Navarro JF, Magnusson J, Giacomello S, Asp M, Westholm JO, Huss M (2016). Visualization and analysis of gene expression in tissue sections by spatial transcriptomics. Science (80-. ).

[62] Rodriques SG, Stickels RR, Goeva A, Martin CA, Murray E, Vanderburg CR, Welch J, Chen LM, Chen F, Macosko EZ (2019). Slide-seq: A scalable technology for measuring genome-wide expression at high spatial resolution. Science (80-. ).

[63] Liu Y, Yang M, Deng Y, Su G, Enninful A, Guo CC, Tebaldi T, Zhang D, Kim D, Bai Z (2020). High-Spatial-Resolution Multi-Omics Sequencing via Deterministic Barcoding in Tissue. Cell.

[64] Lee, J.H. (2017). Quantitative approaches for investigating the spatial context of gene expression. Wiley Interdiscip. Rev Syst Biol Med. 9. 10.1002/wsbm.1369. 10.1002/wsbm.1369 PMC5315614 28001340

[65] Asp M, Bergenstråhle J, Lundeberg J (2020). Spatially Resolved Transcriptomes—Next Generation Tools for Tissue Exploration. BioEssays.

[66] Crosetto N, Bienko M, Van Oudenaarden A (2015). Spatially resolved transcriptomics and beyond. Nat Rev Genet..

[67] Lewis SM, Asselin-Labat ML, Nguyen Q, Berthelet J, Tan X, Wimmer VC, Merino D, Rogers KL, Naik SH (2021). Spatial omics and multiplexed imaging to explore cancer biology. Nat Methods.

[68] Vandereyken K, Sifrim A, Thienpont B, Voet T (2023). Methods and applications for single-cell and spatial multi-omics. Nat Rev Genet..

[69] Rosa M (2008). Fine-needle aspiration biopsy: A historical overview. Diagn Cytopathol..

[70] Bonner, R.F. (1998). Laser Capture Microdissection (LCM) and the Future of Molecular Pathology. Biomed. Opt. Spectrosc. Diagnostics / Ther. Laser Appl. (1998), Pap. JMA2 2, JMA2. 10.1364/AOIPM.1998.JMA2.

[71] Domazet B, MacLennan GT, Lopez-Beltran A, Montironi R, Cheng L (2008). Laser Capture Microdissection in the Genomic and Proteomic Era: Targeting the Genetic Basis of Cancer. Int J Clin Exp Pathol..

[72] Maitra A, Gazdar AF (2001). Tissue microdissection and processing. Cancer Treat Res..

[73] Bryce, A.H., Egan, J.B., Smadbeck, J.B., Johnson, S.H., Murphy, S.J., Harris, F.R., Halling, G.C., Terra, S.B.S.P., Cheville, J., Pagliaro, L., et al. (2019). Shared and unique genomic structural variants of different histological components within testicular germ cell tumours identified with mate pair sequencing. Sci Rep. 9. 10.1038/S41598-019-39956-Y. 10.1038/s41598-019-39956-y PMC6400951 30837548

[74] Ellis P, Moore L, Sanders MA, Butler TM, Brunner SF, Lee-Six H, Osborne R, Farr B, Coorens THH, Lawson ARJ (2020). Reliable detection of somatic mutations in solid tissues by laser-capture microdissection and low-input DNA sequencing. Nat Protoc..

[75] Park ES, Yan JP, Ang RA, Lee JH, Deng X, Duffy SP, Beja K, Annala M, Black PC, Chi KN (2018). Isolation and genome sequencing of individual circulating tumor cells using hydrogel encapsulation and laser capture microdissection. Lab Chip.

[76] Tay, J.K., Zhu, C., Shin, J.H., Zhu, S.X., Varma, S., Foley, J.W., Vennam, S., Yip, Y.L., Goh, C.K., Wang, D.Y., et al. (2022). The microdissected gene expression landscape of nasopharyngeal cancer reveals vulnerabilities in FGF and noncanonical NF-κB signaling. Sci Adv. 8. 10.1126/SCIADV.ABH2445. 10.1126/sciadv.abh2445 PMC8993121 35394843

[77] Tyekucheva, S., Bowden, M., Bango, C., Giunchi, F., Huang, Y., Zhou, C., Bondi, A., Lis, R., Van Hemelrijck, M., Andrén, O., et al. (2017). Stromal and epithelial transcriptional map of initiation progression and metastatic potential of human prostate cancer. Nat Commun. 8. 10.1038/S41467-017-00460-4. 10.1038/s41467-017-00460-4 PMC5583238 28871082

[78] Comba, A., Faisal, S.M., Dunn, P.J., Argento, A.E., Hollon, T.C., Al-Holou, W.N., Varela, M.L., Zamler, D.B., Quass, G.L., Apostolides, P.F., et al. (2022). Spatiotemporal analysis of glioma heterogeneity reveals COL1A1 as an actionable target to disrupt tumor progression. Nat Commun. 13. 10.1038/S41467-022-31340-1. 10.1038/s41467-022-31340-1 PMC9232499 35750880

[79] Liotta, L.A., Pappalardo, P.A., Carpino, A., Haymond, A., Howard, M., Espina, V., Wulfkuhle, J., and Petricoin, E. (2021). Laser Capture Proteomics: spatial tissue molecular profiling from the bench to personalized medicine. 18, 845–861. 10.1080/14789450.2021.1984886 10.1080/14789450.2021.1984886 PMC10720974 34607525

[80] Zhu Y, Dou M, Piehowski PD, Liang Y, Wang F, Chu RK, Chrisler WB, Smith JN, Schwarz KC, Shen Y (2018). Spatially Resolved Proteome Mapping of Laser Capture Microdissected Tissue with Automated Sample Transfer to Nanodroplets. Mol Cell Proteomics.

[81] Yang J, Tong Q, Zhang Y, Yuan S, Gao Y, Deng K, Wang Y, Lu J, Xie X, Zhang Z (2021). Overexpression of Nicotinamide N-methyltransferase mainly covers stroma of colorectal cancer and correlates with unfavorable survival by its product 1-MNA. J Cancer.

[82] Herrera JA, Mallikarjun V, Rosini S, Montero MA, Lawless C, Warwood S, O’Cualain R, Knight D, Schwartz MA, Swift J (2020). Laser capture microdissection coupled mass spectrometry (LCM-MS) for spatially resolved analysis of formalin-fixed and stained human lung tissues. Clin Proteomics.

[83] Ezzoukhry Z, Henriet E, Cordelières FP, Dupuy JW, Maître M, Gay N, Di-Tommaso S, Mercier L, Goetz JG, Peter M (2018). Combining laser capture microdissection and proteomics reveals an active translation machinery controlling invadosome formation. Nat Commun..

[84] Schillebeeckx, M., Schrade, A., Löbs, A.K., Pihlajoki, M., Wilson, D.B., and Mitra, R.D. (2013). Laser capture microdissection-reduced representation bisulfite sequencing (LCM-RRBS) maps changes in DNA methylation associated with gonadectomy-induced adrenocortical neoplasia in the mouse. Nucleic Acids Res. 41. 10.1093/NAR/GKT230. 10.1093/nar/gkt230 PMC3675465 23589626

[85] Meissner A, Gnirke A, Bell GW, Ramsahoye B, Lander ES, Jaenisch R (2005). Reduced representation bisulfite sequencing for comparative high-resolution DNA methylation analysis. Nucleic Acids Res..

[86] Zhao L, Wu X, Zheng J, Dong D (2021). DNA methylome profiling of circulating tumor cells in lung cancer at single base-pair resolution. Oncogene.

[87] Lam KHB, Leon AJ, Hui W, Lee SCE, Batruch I, Faust K, Klekner A, Hutóczki G, Koritzinsky M, Richer M (2022). Topographic mapping of the glioblastoma proteome reveals a triple-axis model of intra-tumoral heterogeneity. Nat Commun.

[88] Zhu, Z., Wang, W., Lin, F., Jordan, T., Li, G., Silverman, S., Qiu, S., Joy, A.A., Chen, C., Hockley, D.L., et al. (2021). Genome profiles of pathologist-defined cell clusters by multiregional LCM and G&T-seq in one triple-negative breast cancer patient. Cell reports Med. 2. 10.1016/J.XCRM.2021.100404. 10.1016/j.xcrm.2021.100404 PMC8561166 34755126

[89] Nitta N, Iino T, Isozaki A, Yamagishi M, Kitahama Y, Sakuma S, Suzuki Y, Tezuka H, Oikawa M, Arai F (2020). Raman image-activated cell sorting. Nat Commun..

[90] Nitta N, Sugimura T, Isozaki A, Mikami H, Hiraki K, Sakuma S, Iino T, Arai F, Endo T, Fujiwaki Y (2018). Intelligent Image-Activated Cell Sorting. Cell.

[91] Lee, S., Lee, W., Lee, A.C., Nam, J., Lee, J., Kim, H., and Lee, S. (2022). I-LIFT (image-based laser-induced forward transfer) platform for manipulating encoded microparticles. Biomicrofluidics 061101. 10.1063/5.0131733. 10.1063/5.0131733 PMC9726220 36483021

[92] Kim S, Lee AC, Lee HB, Kim J, Jung Y, Ryu HS, Lee Y, Bae S, Lee M, Lee K (2018). PHLI-seq: constructing and visualizing cancer genomic maps in 3D by phenotype-based high-throughput laser-aided isolation and sequencing. Genome Biol..

[93] Kim J, Kim S, Yeom H, Song SW, Shin K, Bae S, Ryu HS, Kim JY, Choi A, Lee S (2023). Barcoded multiple displacement amplification for high coverage sequencing in spatial genomics. Nat Commun..

[94] Kim O, Lee D, Chungwon Lee A, Lee Y, Bae HJ, Lee HB, Kim RN, Han W, Kwon S (2019). Whole Genome Sequencing of Single Circulating Tumor Cells Isolated by Applying a Pulsed Laser to Cell-Capturing Microstructures. Small.

[95] Lee AC, Lee Y, Choi A, Lee HB, Shin K, Lee H, Kim JY, Ryu HS, Kim HS, Ryu SY (2022). Spatial epitranscriptomics reveals A-to-I editome specific to cancer stem cell microniches. Nat. Commun..

[96] Jeong D, Lee AC, Shin K, Kim J, Ham MH, Lee C, Lee S, Choi A, Ryu T, Kim O (2023). Hema-seq reveals genomic aberrations in a rare simultaneous occurrence of hematological malignancies. Cell Rep Methods.

[97] Kulkarni, M.M. (2011). Digital Multiplexed Gene Expression Analysis Using the NanoString nCounter System. Curr Protoc Mol Biol. 94, 25B.10.1-25B.10.17. 10.1002/0471142727.MB25B10S94. 10.1002/0471142727.mb25b10s94 21472696

[98] Hernandez S, Lazcano R, Serrano A, Powell S, Kostousov L, Mehta J, Khan K, Lu W, Solis LM (2022). Challenges and Opportunities for Immunoprofiling Using a Spatial High-Plex Technology: The NanoString GeoMx® Digital Spatial Profiler. Front. Oncol..

[99] Han S, Fu D, Tushoski GW, Meng L, Herremans KM, Riner AN, Geoge TJ, Huo Z, Hughes SJ (2022). Single-cell profiling of microenvironment components by spatial localization in pancreatic ductal adenocarcinoma. Theranostics.

[100] Giesen C, Wang HAO, Schapiro D, Zivanovic N, Jacobs A, Hattendorf B, Schüffler PJ, Grolimund D, Buhmann JM, Brandt S (2014). Highly multiplexed imaging of tumor tissues with subcellular resolution by mass cytometry. Nat. Methods.

[101] Keren, L., Bosse, M., Thompson, S., Risom, T., Vijayaragavan, K., McCaffrey, E., Marquez, D., Angoshtari, R., Greenwald, N.F., Fienberg, H., et al. (2019). MIBI-TOF: A multiplexed imaging platform relates cellular phenotypes and tissue structure. Sci Adv. 5. 10.1126/SCIADV.AAX5851. 10.1126/sciadv.aax5851 PMC6785247 31633026

[102] Mi, H., Ho, W.J., Yarchoan, M., and Popel, A.S. (2022). Multi-Scale Spatial Analysis of the Tumor Microenvironment Reveals Features of Cabozantinib and Nivolumab Efficacy in Hepatocellular Carcinoma. Front Immunol. 13. 10.3389/FIMMU.2022.892250. 10.3389/fimmu.2022.892250 PMC9136005 35634309

[103] Ji, A.L., Rubin, A.J., Thrane, K., Jiang, S., Reynolds, D.L., Meyers, R.M., Guo, M.G., George, B.M., Mollbrink, A., Bergenstråhle, J., et al. (2020). Multimodal Analysis of Composition and Spatial Architecture in Human Squamous Cell Carcinoma. J Clean Prod., 497–514. 10.1016/j.cell.2020.05.039. 10.1016/j.cell.2020.05.039 PMC7391009 32579974

[104] Zhu Q, Shah S, Dries R, Cai L, Yuan GC (2018). Identification of spatially associated subpopulations by combining scRNAseq and sequential fluorescence in situ hybridization data. Nat Biotechnol..

[105] Lewis SM, Asselin-Labat ML, Nguyen Q, Berthelet J, Tan X, Wimmer VC, Merino D, Rogers KL, Naik SH (2021). Spatial omics and multiplexed imaging to explore cancer biology. Nat Methods.

[106] Li Q, Zhang X, Ke R (2022). Spatial Transcriptomics for Tumor Heterogeneity Analysis. Front Genet..

[107] Marx V (2021). Method of the Year: spatially resolved transcriptomics. Nat Methods.

[108] Singer RH, Ward DC (1982). Actin gene expression visualized in chicken muscle tissue culture by using in situ hybridization with a biotinated nucleotide analog. Proc Natl Acad Sci..

[109] Femino AM, Fay FS, Fogarty K, Singer RH (1998). Visualization of single RNA transcripts in situ. Science (80-. ).

[110] Gall JG, Pardue ML (1969). Formation and detection of RNA-DNA hybrid molecules in cytological preparations. Proc Natl Acad Sci U S A.

[111] Levsky JM, Shenoy SM, Pezo RC, Singer RH (2002). Single-cell gene expression profiling. Science (80-. ).

[112] Dirks, R.W., Van Gijlswijk, R.P.M., Vooijs, M.A., Smit, A.B., Bogerd, ~ J, Van Minnen, ? J, Raap, ~ A K, and Van Der Ploeg, M. (1991). 3’-End Fluorochromized and Haptenized Oligonucleotides as in Situ Hybridization Probes for Multiple, Simultaneous RNA Detection’. 10.1016/0014-4827(91)90370-a 2026182

[113] Raj A, van den Bogaard P, Rifkin SA, van Oudenaarden A, Tyagi S (2008). Imaging individual mRNA molecules using multiple singly labeled probes. Nat. Methods.

[114] Femino AM, Fay FS, Fogarty K, Singer RH (1998). Visualization of single RNA transcripts in situ. Science (80-. ).

[115] Wang F, Flanagan J, Su N, Wang LC, Bui S, Nielson A, Wu X, Vo HT, Ma XJ, Luo Y (2012). RNAscope: A Novel in Situ RNA Analysis Platform for Formalin-Fixed Paraffin-Embedded Tissues. J Mol Diagnostics.

[116] Chen M, Andreozzi M, Pockaj B, Barrett MT, Ocal IT, McCullough AE, Linnaus ME, Chang JM, Yearley JH, Annamalai L (2017). Development and validation of a novel clinical fluorescence in situ hybridization assay to detect JAK2 and PD-L1 amplification: a fluorescence in situ hybridization assay for JAK2 and PD-L1 amplification. Mod Pathol..

[117] Borazanci E, Millis SZ, Kimbrough J, Doll N, von Hoff D, Ramanathan RK (2017). Potential actionable targets in appendiceal cancer detected by immunohistochemistry, fluorescent in situ hybridization, and mutational analysis. J Gastrointest Oncol..

[118] Zakrzewski F, de Back W, Weigert M, Wenke T, Zeugner S, Mantey R, Sperling C, Friedrich K, Roeder I, Aust D (2019). Automated detection of the HER2 gene amplification status in Fluorescence in situ hybridization images for the diagnostics of cancer tissues. Sci. Reports.

[119] Annaratone L, Simonetti M, Wernersson E, Marchiò C, Garnerone S, Scalzo MS, Bienko M, Chiarle R, Sapino A, Crosetto N (2017). Quantification of HER2 and estrogen receptor heterogeneity in breast cancer by single-molecule RNA fluorescence in situ hybridization. Oncotarget.

[120] Rowland TJ, Dumbović G, Hass EP, Rinn JL, Cech TR (2019). Single-cell imaging reveals unexpected heterogeneity of telomerase reverse transcriptase expression across human cancer cell lines. Proc Natl Acad. Sci U S A..

[121] Lubeck E, Coskun AF, Zhiyentayev T, Ahmad M, Cai L (2014). Single cell in situ RNA profiling by sequentialhybridization. Nat Methods.

[122] Eng C-HL, Lawson M, Zhu Q, Dries R, Koulena N, Takei Y, Yun J, Cronin C, Karp C, Yuan G-C (2019). Transcriptome-scale super-resolved imaging in tissues by RNA seqFISH+. Nat..

[123] Xia C, Babcock HP, Moffitt JR, Zhuang X (2019). Multiplexed detection of RNA using MERFISH and branched DNA amplification. Sci Rep.

[124] Hara T, Chanoch-Myers R, Mathewson ND, Myskiw C, Atta L, Bussema L, Eichhorn SW, Greenwald AC, Kinker GS, Rodman C (2021). Interactions between cancer cells and immune cells drive transitions to mesenchymal-like states in glioblastoma. Cancer Cell.

[125] Gyllborg D, Langseth CM, Qian X, Choi E, Salas SM, Hilscher MM, Lein ES, Nilsson M (2020). Hybridization-based in situ sequencing (HybISS) for spatially resolved transcriptomics in human and mouse brain tissue. Nucleic Acids Res..

[126] Krzywkowski T, Kühnemund M, Nilsson M (2019). Chimeric padlock and iLock probes for increased efficiency of targeted RNA detection. Rna.

[127] Lee H, Marco Salas S, Gyllborg D, Nilsson M (2022). Direct RNA targeted in situ sequencing for transcriptomic profiling in tissue. Sci Reports.

[128] Svedlund J, Strell C, Qian X, Zilkens KJC, Tobin NP, Bergh J, Sieuwerts AM, Nilsson M (2019). Generation of in situ sequencing based OncoMaps to spatially resolve gene expression profiles of diagnostic and prognostic markers in breast cancer. EBioMedicine.

[129] Chen F, Tillberg PW, Boyden ES (2015). Expansion microscopy. Science (80-. ).

[130] Alon, S., Goodwin, D.R., Sinha, A., Wassie, A.T., Chen, F., Daugharthy, E.R., Bando, Y., Kajita, A., Xue, A.G., Marrett, K., et al. (2021). Expansion sequencing: Spatially precise in situ transcriptomics in intact biological systems. Science 371. 10.1126/SCIENCE.AAX2656. 10.1126/science.aax2656 PMC7900882 33509999

[131] Wang, X., Allen, W.E., Wright, M.A., Sylwestrak, E.L., Samusik, N., Vesuna, S., Evans, K., Liu, C., Ramakrishnan, C., Liu, J., et al. (2018). Three-dimensional intact-tissue sequencing of single-cell transcriptional states. Science 361. 10.1126/SCIENCE.AAT5691. 10.1126/science.aat5691 PMC6339868 29930089

[132] Vickovic S, Eraslan G, Salmén F, Klughammer J, Stenbeck L, Schapiro D, Äijö T, Bonneau R, Bergenstråhle L, Navarro JF (2019). High-definition spatial transcriptomics for in situ tissue profiling. Nat Methods.

[133] Andersson A, Larsson L, Stenbeck L, Salmén F, Ehinger A, Wu SZ, Al-Eryani G, Roden D, Swarbrick A, Borg Å (2021). Spatial deconvolution of HER2-positive breast cancer delineates tumor-associated cell type interactions. Nat Commun..

[134] Andersson A, Bergenstråhle J, Asp M, Bergenstråhle L, Jurek A, Fernández Navarro J, Lundeberg J (2020). Single-cell and spatial transcriptomics enables probabilistic inference of cell type topography. Commun Biol..

[135] Booeshaghi AS, Yao Z, van Velthoven C, Smith K, Tasic B, Zeng H, Pachter L (2021). Isoform cell-type specificity in the mouse primary motor cortex. Nat..

[136] K, L., J, B., K, T., A, M., K, M., P, B., R, W., and J, L. (2020). The spatial landscape of gene expression isoforms in tissue sections10.1101/2020.08.24.252296

[137] Boileau, E., Li, X., Vries, I.S.N., Becker, C., Casper, R., Altmüller, J., Leuschner, F., and Dieterich, C. (2022). Full-Length Spatial Transcriptomics Reveals the Unexplored Isoform Diversity of the Myocardium Post-MI. Front. Genet. 13. 10.3389/FGENE.2022.912572. 10.3389/fgene.2022.912572 PMC9354982 35937994

[138] Erickson A, He M, Berglund E, Marklund M, Mirzazadeh R, Schultz N, Kvastad L, Andersson A, Bergenstråhle L, Bergenstråhle J (2022). Spatially resolved clonal copy number alterations in benign and malignant tissue. Nat..

[139] Wu SZ, Al-Eryani G, Roden DL, Junankar S, Harvey K, Andersson A, Thennavan A, Wang C, Torpy JR, Bartonicek N (2021). A single-cell and spatially resolved atlas of human breast cancers. Nat Genet..

[140] Thrane K, Eriksson H, Maaskola J, Hansson J, Lundeberg J (2018). Spatially resolved transcriptomics enables dissection of genetic heterogeneity in stage III cutaneous malignant melanoma. Cancer Res..

[141] Sun H, Zhang D, Huang C, Guo Y, Yang Z, Yao N, Dong X, Cheng R, Zhao N, Meng J (2021). Hypoxic microenvironment induced spatial transcriptome changes in pancreatic cancer. Cancer Biol Med..

[142] Moncada R, Barkley D, Wagner F, Chiodin M, Devlin JC, Baron M, Hajdu CH, Simeone DM, Yanai I (2020). Integrating microarray-based spatial transcriptomics and single-cell RNA-seq reveals tissue architecture in pancreatic ductal adenocarcinomas. Nat Biotechnol..

[143] Wu Y, Yang S, Ma J, Chen Z, Song G, Rao D, Cheng Y, Huang S, Liu Y, Jiang S (2022). Spatiotemporal Immune Landscape of Colorectal Cancer Liver Metastasis at Single-Cell Level. Cancer Discov..

[144] Ji AL, Rubin AJ, Thrane K, Jiang S, Reynolds DL, Meyers RM, Guo MG, George BM, Mollbrink A, Bergenstråhle J (2020). Multimodal Analysis of Composition and Spatial Architecture in Human Squamous Cell Carcinoma. Cell.

[145] Ravi VM, Neidert N, Will P, Joseph K, Maier JP, Kückelhaus J, Vollmer L, Goeldner JM, Behringer SP, Scherer F (2022). T-cell dysfunction in the glioblastoma microenvironment is mediated by myeloid cells releasing interleukin-10. Nat Commun..

[146] Nieto P, Elosua-Bayes M, Trincado JL, Marchese D, Massoni-Badosa R, Salvany M, Henriques A, Nieto J, Aguilar-Fernández S, Mereu E (2021). A single-cell tumor immune atlas for precision oncology. Genome Res..

[147] Hunter MV, Moncada R, Weiss JM, Yanai I, White RM (2021). Spatially resolved transcriptomics reveals the architecture of the tumor-microenvironment interface. Nat Commun..

[148] Chen A, Liao S, Cheng M, Ma K, Wu L, Lai Y, Qiu X, Yang J, Xu J, Hao S (2022). Spatiotemporal transcriptomic atlas of mouse organogenesis using DNA nanoball-patterned arrays. Cell.

[149] Parra I, Windle B (1993). High resolution visual mapping of stretched DNA by fluorescent hybridization. Nat Genet..

[150] Levsky JM, Singer RH (2003). Fluorescence in situ hybridization: past, present and future. J Cell Sci..

[151] Nederlof PM, van der Flier S, Wiegant J, Raap AK, Tanke HJ, Ploem JS, van der Ploeg M (1990). Multiple fluorescence in situ hybridization. Cytometry.

[152] Bauman JGJ, Wiegant J, Borst P, van Duijn P (1980). A new method for fluorescence microscopical localization of specific DNA sequences by in situ hybridization of fluorochromelabelled RNA. Exp Cell Res..

[153] Beliveau BJ, Boettiger AN, Avendaño MS, Jungmann R, McCole RB, Joyce EF, Kim-Kiselak C, Bantignies F, Fonseka CY, Erceg J (2015). Single-molecule super-resolution imaging of chromosomes and in situ haplotype visualization using Oligopaint FISH probes. Nat Commun..

[154] Payne, A.C., Chiang, Z.D., Reginato, P.L., Mangiameli, S.M., Murray, E.M., Yao, C.C., Markoulaki, S., Earl, A.S., Labade, A.S., Jaenisch, R., et al. (2021). In situ genome sequencing resolves DNA sequence and structure in intact biological samples. Science (80-. ). 371. 10.1126/SCIENCE.AAY3446/SUPPL_FILE/AAY3446-PAYNE-SM.PDF. 10.1126/science.aay3446 PMC7962746 33384301

[155] Zhao T, Chiang ZD, Morriss JW, LaFave LM, Murray EM, Del Priore I, Meli K, Lareau CA, Nadaf NM, Li J (2021). Spatial genomics enables multi-modal study of clonal heterogeneity in tissues. Nat..

[156] Kaya-Okur HS, Wu SJ, Codomo CA, Pledger ES, Bryson TD, Henikoff JG, Ahmad K, Henikoff S (2019). CUT&Tag for efficient epigenomic profiling of small samples and single cells. Nat Commun..

[157] Thornton CA, Mulqueen RM, Torkenczy KA, Nishida A, Lowenstein EG, Fields AJ, Steemers FJ, Zhang W, McConnell HL, Woltjer RL (2021). Spatially mapped single-cell chromatin accessibility. Nat. Commun..

[158] Ernst J, Kheradpour P, Mikkelsen TS, Shoresh N, Ward LD, Epstein CB, Zhang X, Wang L, Issner R, Coyne M (2011). Mapping and analysis of chromatin state dynamics in nine human cell types. Nat..

[159] Deng Y, Bartosovic M, Kukanja P, Zhang D, Liu Y, Su G, Enninful A, Bai Z, Castelo-Branco G, Fan R (2022). Spatial-CUT&Tag: Spatially resolved chromatin modification profiling at the cellular level. Science.

[160] Deng Y, Bartosovic M, Ma S, Zhang D, Kukanja P, Xiao Y, Su G, Liu Y, Qin X, Rosoklija GB (2022). Spatial profiling of chromatin accessibility in mouse and human tissues. Nat..

[161] Lu T, Ang CE, Zhuang X (2022). Spatially resolved epigenomic profiling of single cells in complex tissues. Cell.

[162] Fan, R., Zhang, D., Deng, Y., Kukanja, P., Bartosovic, M., Institutet, K., Su, G., Bao, S., Liu, Y., Xiao, Y., et al. Spatially resolved epigenome-transcriptome co-proling of mammalian tissues at the cellular level. Preprint. 10.21203/rs.3.rs-1728747/v1.

[163] Banik G, Betts CB, Liudahl SM, Sivagnanam S, Kawashima R, Cotechini T, Larson W, Goecks J, Pai SI, Clayburgh DR (2020). High-dimensional multiplexed immunohistochemical characterization of immune contexture in human cancers. Methods Enzymol..

[164] Tsujikawa T, Kumar S, Borkar RN, Azimi V, Thibault G, Chang YH, Balter A, Kawashima R, Choe G, Sauer D (2017). Quantitative Multiplex Immunohistochemistry Reveals Myeloid-Inflamed Tumor-Immune Complexity Associated with Poor Prognosis. Cell Rep..

[165] Tóth ZE, Mezey É (2007). Simultaneous visualization of multiple antigens with tyramide signal amplification using antibodies from the same species. J Histochem Cytochem..

[166] Goltsev Y, Samusik N, Kennedy-Darling J, Bhate S, Hale M, Vazquez G, Black S, Nolan GP (2018). Deep Profiling of Mouse Splenic Architecture with CODEX Multiplexed Imaging. Cell.

[167] Black S, Phillips D, Hickey JW, Kennedy-Darling J, Venkataraaman VG, Samusik N, Goltsev Y, Schürch CM, Nolan GP (2021). CODEX multiplexed tissue imaging with DNA-conjugated antibodies. Nat Protoc..

[168] Lu S, Stein JE, Rimm DL, Wang DW, Bell JM, Johnson DB, Sosman JA, Schalper KA, Anders RA, Wang H (2019). Comparison of Biomarker Modalities for Predicting Response to PD-1/PD-L1 Checkpoint Blockade: A Systematic Review and Meta-analysis. JAMA Oncol..

[169] Phillips, D., Schürch, C.M., Khodadoust, M.S., Kim, Y.H., Nolan, G.P., and Jiang, S. (2021). Highly Multiplexed Phenotyping of Immunoregulatory Proteins in the Tumor Microenvironment by CODEX Tissue Imaging. Front. Immunol. 12. 10.3389/FIMMU.2021.687673/FULL. 10.3389/fimmu.2021.687673 PMC8170307 34093591

[170] Chakiryan NH, Hajiran A, Kim Y, Aydin AM, Zemp L, Katende E, Nguyen J, Fan W, Cheng CH, Lopez-Blanco N (2022). Correlating Immune Cell Infiltration Patterns with Recurrent Somatic Mutations in Advanced Clear Cell Renal Cell Carcinoma. Eur Urol Focus.

[171] de Andrea CE, Ochoa MC, Villalba-Esparza M, Teijeira Á, Schalper KA, Abengozar-Muela M, Eguren-Santamaría I, Sainz C, Sánchez-Gregorio S, Garasa S (2021). Heterogenous presence of neutrophil extracellular traps in human solid tumours is partially dependent on IL-8. J Pathol..

[172] Mondello P, Fama A, Larson MC, Feldman AL, Villasboas JC, Yang ZZ, Galkin I, Svelolkin V, Postovalova E, Bagaev A (2021). Lack of intrafollicular memory CD4 + T cells is predictive of early clinical failure in newly diagnosed follicular lymphoma. Blood Cancer J..

[173] Lundberg, E., and Borner, G.H.H. Spatial proteomics: a powerful discovery tool for cell biology. Nat Rev Mol Cell Biol. 10.1038/s41580-018-0094-y. 10.1038/s41580-018-0094-y 30659282

[174] Hasin Y, Seldin M, Lusis A (2017). Multi-omics approaches to disease. Genome Biol..

[175] Stoeckius M, Hafemeister C, Stephenson W, Houck-Loomis B, Chattopadhyay PK, Swerdlow H, Satija R, Smibert P (2017). Simultaneous epitope and transcriptome measurement in single cells. Nat Methods.

[176] Vickovic S, Lötstedt B, Klughammer J, Mages S, Segerstolpe, Rozenblatt-Rosen O, Regev A. SM-Omics is an automated platform for high-throughput spatial multi-omics. Nat. Commun. 2022;131(13):1–13. 10.1038/s41467-022-28445-y. 10.1038/s41467-022-28445-y PMC8831571 35145087

[177] Zhang S, Deshpande A, Verma BK, Wang H, Mi H, Yuan L, Ho WJ, Jaffee EM, Zhu Q, Anders RA (2023). Informing virtual clinical trials of hepatocellular carcinoma with spatial multi-omics analysis of a human neoadjuvant immunotherapy clinical trial. bioRxiv.

[178] Ruiz-Martinez A, Gong C, Wang H, Sove RJ, Mi H, Kimko H, Popel AS (2022). Simulations of tumor growth and response to immunotherapy by coupling a spatial agent-based model with a whole-patient quantitative systems pharmacology model. PLOS Comput Biol..

[179] Song AH, Jaume G, Williamson DFK, Lu MY, Vaidya A, Miller TR, Mahmood F (2023). Artificial intelligence for digital and computational pathology. Nat Rev Bioeng..

[180] Tuong ZK, Loudon KW, Berry B, Richoz N, Jones J, Tan X, Nguyen Q, George A, Hori S, Field S (2021). Resolving the immune landscape of human prostate at a single-cell level in health and cancer. Cell Rep..

[181] Gouin KH, Ing N, Plummer JT, Rosser CJ, Ben Cheikh B, Oh C, Chen SS, Chan KS, Furuya H, Tourtellotte WG (2021). An N-Cadherin 2 expressing epithelial cell subpopulation predicts response to surgery, chemotherapy and immunotherapy in bladder cancer. Nat Commun..

[182] Joseph DB, Henry GH, Malewska A, Reese JC, Mauck RJ, Gahan JC, Hutchinson RC, Mohler JL, Roehrborn CG, Strand DW (2022). 5-Alpha reductase inhibitors induce a prostate luminal to club cell transition in human benign prostatic hyperplasia. J Pathol..

[183] Luca BA, Steen CB, Matusiak M, Azizi A, Varma S, Zhu C, Przybyl J, Espín-Pérez A, Diehn M, Alizadeh AA (2021). Atlas of clinically distinct cell states and ecosystems across human solid tumors. Cell.

[184] Ma Y, Zhou X (2022). Spatially informed cell-type deconvolution for spatial transcriptomics. Nat Biotechnol..

[185] van de Velde LA, Kaitlynn Allen E, Crawford JC, Wilson TL, Guy CS, Russier M, Zeitler L, Bahrami A, Finkelstein D, Pelletier S (2021). Neuroblastoma formation requires unconventional CD4 T cells and arginase-1–dependent myeloid cells. Cancer Res..

[186] Dhainaut M, Rose SA, Akturk G, Wroblewska A, Nielsen SR, Park ES, Buckup M, Roudko V, Pia L, Sweeney R (2022). Spatial CRISPR genomics identifies regulators of the tumor microenvironment. Cell.

[187] Wei R, He S, Bai S, Sei E, Hu M, Thompson A, Chen K, Krishnamurthy S, Navin NE (2022). Spatial charting of single-cell transcriptomes in tissues. Nat Biotechnol..

[188] Magen A, Hamon P, Fiaschi N, Troncoso L, Humblin E, D’souza D, Dawson T, Park MD, Kim J, Hamel S (2022). Intratumoral mregDC and CXCL13 T helper niches enable local differentiation of CD8 T cells following PD-1 blockade. BioRxiv.

[189] Ruiz-Moreno C, Salas SM, Samuelsson E, Brandner S, Kranendonk MEG, Nilsson M, Stunnenberg HG (2022). Harmonized single-cell landscape, intercellular crosstalk and tumor architecture of glioblastoma. bioRxiv.

[190] Alon S, Goodwin DR, Sinha A, Wassie AT, Chen F, Daugharthy ER, Bando Y, Kajita A, Xue AG, Marrett K (2021). Expansion sequencing: Spatially precise in situ transcriptomics in intact biological systems. Science (80-. ).

[191] Tamma R, Annese T, Ruggieri S, Marzullo A, Nico B, Ribatti D (2018). VEGFA and VEGFR2 RNAscope determination in gastric cancer. J. Mol. Histol..

[192] Zagozewski, J., Borlase, S., Guppy, B.J., Coudière-Morrison, L., Shahriary, G.M., Gordon, V., Liang, L., Cheng, S., Porter, C.J., Kelley, R., et al. (2022). Combined MEK and JAK/STAT3 pathway inhibition effectively decreases SHH medulloblastoma tumor progression. Commun Biol. 5. 10.1038/S42003-022-03654-9. 10.1038/s42003-022-03654-9 PMC9283517 35835937

[193] Brady L, Kriner M, Coleman I, Morrissey C, Roudier M, True LD, Gulati R, Plymate SR, Zhou Z, Birditt B (2021). Inter- and intra-tumor heterogeneity of metastatic prostate cancer determined by digital spatial gene expression profiling. Nat Commun..

[194] Kulasinghe, A., Monkman, J., Shah, E.T., Matigian, N., Adams, M.N., and O’Byrne, K. (2021). Spatial Profiling Identifies Prognostic Features of Response to Adjuvant Therapy in Triple Negative Breast Cancer (TNBC). Front Oncol. 11. 10.3389/FONC.2021.798296. 10.3389/fonc.2021.798296 PMC8784863 35083152

[195] Sharma A, Seow JJW, Dutertre CA, Pai R, Blériot C, Mishra A, Wong RMM, Singh GSN, Sudhagar S, Khalilnezhad S (2020). Onco-fetal Reprogramming of Endothelial Cells Drives Immunosuppressive Macrophages in Hepatocellular Carcinoma. Cell.

[196] Pelka K, Hofree M, Chen JH, Sarkizova S, Pirl JD, Jorgji V, Bejnood A, Dionne D, Ge WH, Xu KH (2021). Spatially organized multicellular immune hubs in human colorectal cancer. Cell.

[197] van Krimpen A, Gerretsen VIV, Mulder EEAP, van Gulijk M, van den Bosch TPP, von der Thüsen J, Grünhagen DJ, Verhoef C, Mustafa D, Aerts JG (2022). Immune suppression in the tumor-draining lymph node corresponds with distant disease recurrence in patients with melanoma. Cancer Cell.

[198] Wong-Rolle, A., Dong, Q., Zhu, Y., Divakar, P., Hor, J.L., Kedei, N., Wong, M., Tillo, D., Conner, E.A., Rajan, A., et al. (2022). Spatial meta-transcriptomics reveal associations of intratumor bacteria burden with lung cancer cells showing a distinct oncogenic signature. J Immunother Cancer 10. 10.1136/JITC-2022-004698. 10.1136/jitc-2022-004698 PMC9260850 35793869

[199] Han S, Fu D, Tushoski GW, Meng L, Herremans KM, Riner AN, Geoge TJ, Huo Z, Hughes SJ (2022). Single-cell profiling of microenvironment components by spatial localization in pancreatic ductal adenocarcinoma. Theranostics.

[200] Sadeghirad, H., Monkman, J., Mehdi, A.M., Ladwa, R., O’Byrne, K., Hughes, B.G.M., and Kulasinghe, A. (2022). Dissecting Tissue Compartment-Specific Protein Signatures in Primary and Metastatic Oropharyngeal Squamous Cell Carcinomas. Front Immunol. 13. 10.3389/FIMMU.2022.895513. 10.3389/fimmu.2022.895513 PMC9149425 35651606

[201] Schmitd LB, Perez-Pacheco C, Bellile EL, Wu W, Casper K, Mierzwa M, Rozek LS, Wolf GT, Taylor JMG, D’Silva NJ (2022). Spatial and Transcriptomic Analysis of Perineural Invasion in Oral Cancer. Clin. Cancer Res..

[202] McNamara KL, Caswell-Jin JL, Joshi R, Ma Z, Kotler E, Bean GR, Kriner M, Zhou Z, Hoang M, Beechem J (2021). Spatial proteomic characterization of HER2-positive breast tumors through neoadjuvant therapy predicts response. Nat Cancer.

[203] Zhang L, Li Z, Skrzypczynska KM, Fang Q, Zhang W, O’Brien SA, He Y, Wang L, Zhang Q, Kim A (2020). Single-Cell Analyses Inform Mechanisms of Myeloid-Targeted Therapies in Colon Cancer. Cell.

[204] Che LH, Liu JW, Huo JP, Luo R, Xu RM, He C, Li YQ, Zhou AJ, Huang P, Chen YY (2021). A single-cell atlas of liver metastases of colorectal cancer reveals reprogramming of the tumor microenvironment in response to preoperative chemotherapy. Cell Discov.

[205] Pombo Antunes AR, Scheyltjens I, Lodi F, Messiaen J, Antoranz A, Duerinck J, Kancheva D, Martens L, De Vlaminck K, Van Hove H (2021). Single-cell profiling of myeloid cells in glioblastoma across species and disease stage reveals macrophage competition and specialization. Nat Neurosci..

[206] Mi H, Sivagnanam S, Betts CB, Liudahl SM, Jaffee EM, Coussens LM, Popel AS (2022). Quantitative Spatial Profiling of Immune Populations in Pancreatic Ductal Adenocarcinoma Reveals Tumor Microenvironment Heterogeneity and Prognostic Biomarkers. Cancer Res..

[207] Liudahl SM, Betts CB, Sivagnanam S, Morales-Oyarvide V, Silva A. Da, Yuan C, Hwang S, Grossblatt-Wait A, Leis KR, Larson W (2021). Leukocyte heterogeneity in pancreatic ductal adenocarcinoma: Phenotypic and spatial features associated with clinical outcome. Cancer Discov..

[208] Mi H, Bivalacqua TJ, Kates M, Seiler R, Black PC, Popel AS, Baras AS (2021). Predictive models of response to neoadjuvant chemotherapy in muscle-invasive bladder cancer using nuclear morphology and tissue architecture. Cell Reports Med..

[209] Nakhli, R., Moghadam, P.A., Mi, H., Farahani, H., Baras, A., Gilks, B., and Bashashati, A. (2023). Sparse Multi-Modal Graph Transformer with Shared-Context Processing for Representation Learning of Giga-pixel Images. 2023 IEEE/CVF Conf. Comput. Vis. Pattern Recognit. 2023-June, 11547–11557. 10.1109/CVPR52729.2023.01111.

[210] Schürch CM, Bhate SS, Barlow GL, Phillips DJ, Noti L, Zlobec I, Chu P, Black S, Demeter J, McIlwain DR (2020). Coordinated Cellular Neighborhoods Orchestrate Antitumoral Immunity at the Colorectal Cancer Invasive Front. Cell.

[211] Phillips D, Matusiak M, Gutierrez BR, Bhate SS, Barlow GL, Jiang S, Demeter J, Smythe KS, Pierce RH, Fling SP (2021). Immune cell topography predicts response to PD-1 blockade in cutaneous T cell lymphoma. Nat Commun..

[212] Cato L, de Tribolet-Hardy J, Lee I, Rottenberg JT, Coleman I, Melchers D, Houtman R, Xiao T, Li W, Uo T (2019). ARv7 Represses Tumor-Suppressor Genes in Castration-Resistant Prostate Cancer. Cancer Cell.

[213] Baldelli E, Mandarano M, Bellezza G, Petricoin EF, Pierobon M (2022). Analysis of neuroendocrine clones in NSCLCs using an immuno-guided laser-capture microdissection-based approach. Cell Rep Methods.

[214] Gómez-Cuadrado L, Bullock E, Mabruk Z, Zhao H, Souleimanova M, Noer PR, Turnbull AK, Oxvig C, Bertos N, Byron A (2022). Characterisation of the Stromal Microenvironment in Lobular Breast Cancer. Cancers.

[215] Chowdhuri SR, Xi L, Pham THT, Hanson J, Rodriguez-Canales J, Berman A, Rajan A, Giaccone G, Emmert-Buck M, Raffeld M (2012). EGFR and KRAS mutation analysis in cytologic samples of lung adenocarcinoma enabled by laser capture microdissection. Mod Pathol..

[216] Malapelle U, De Rosa N, Rocco D, Bellevicine C, Crispino C, Illiano A, Piantedosi FV, Nappi O, Troncone G (2012). EGFR and KRAS mutations detection on lung cancer liquid-based cytology: a pilot study. J Clin Pathol..

[217] Tay J, Teo WK, Goh CK, Wu BC, Loh KS (2022). Abstract 774: Microdissected gene expression profiling of recurrent nasopharyngeal carcinoma. Cancer Res..

[218] Rubin MA, Gerstein A, Reid K, Bostwick DG, Cheng L, Parsons R, Papadopoulos N (2000). 1Oq23.3 loss of heterozygosity is higher inlymph node-positive (PT2-3, N+) versus lymph node-negative (PT2-3, N0) prostate cancer. Hum Pathol..

[219] Cheng L, Bostwick DG, Li G, Zhang S, Vortmeyer AO, Zhuang Z (2001). Conserved Genetic Findings in Metastatic Bladder CancerA Possible Utility of Allelic Loss of Chromosomes 9p21 and 17p13 in Diagnosis. Arch Pathol Lab Med..

[220] Bertheau P, Plassa LF, Lerebours F, De Roquancourt A, Turpin E, Lidereau R, De Thé H, Janin A (2001). Allelic Loss Detection in Inflammatory Breast Cancer: Improvement with Laser Microdissection. Lab Investig..

[221] Cheng L, MacLennan GT, Pan CX, Jones TD, Moore CR, Zhang S, Gu J, Patel NB, Kao C, Gardner TA (2004). Allelic Loss of the Active X Chromosome During Bladder Carcinogenesis. Arch Pathol Lab Med..

[222] Zhang X, Leav I, Revelo MP, Deka R, Medvedovic M (2009). Deletion Hotspots in AMACR Promoter CpG Island Are cis-Regulatory Elements Controlling the Gene Expression in the Colon. PLoS Genet.

[223] Zhu X, Wen S, Deng S, Wu G, Tian R, Hu P, Ye L, Sun Q, Xu Y, Deng G (2022). A Novel Karyoplasmic Ratio-Based Automatic Recognition Method for Identifying Glioma Circulating Tumor Cells. Front Oncol..

[224] Wild P, Knuechel R, Dietmaier W, Hofstaedter F, Hartmann A (2000). Laser Microdissection and Microsatellite Analyses of Breast Cancer Reveal a High Degree of Tumor Heterogeneity. Pathobiology.

[225] Cheng L, Gu J, Ulbright TM, MacLennan GT, Sweeney CJ, Zhang S, Sanchez K, Koch MO, Eble JN (2002). Precise microdissection of human bladder carcinomas reveals divergent tumor subclones in the same tumor. Cancer.

[226] Jones TD, Eble JN, Wang M, MacLennan GT, Jain S, Cheng L (2005). Clonal divergence and genetic heterogeneity in clear cell renal cell carcinomas with sarcomatoid transformation. Cancer.

[227] Katona TM, Jones TD, Wang M, Eble JN, Billings SD, Cheng L (2007). Genetically heterogeneous and clonally unrelated metastases may arise in patients with cutaneous melanoma. Am J Surg Pathol..

[228] Olafsson S, Anderson CA (2021). Somatic mutations provide important and unique insights into the biology of complex diseases. Trends Genet..

[229] Cowherd SM, Espina VA, Petricoin EF, Liotta LA (2004). Proteomic Analysis of Human Breast Cancer Tissue with Laser-Capture Microdissection and Reverse-Phase Protein Microarrays. Clin Breast Cancer.

[230] Buckanovich R, Jenkins A, Katsaros D, Buckanovich RJ, Sasaroli D, O’brien-Jenkins, A., Botbyl, J., Hammond, R., Katsaros, D., Sandaltzopoulos, R.,  (2007). Tumor Vascular Proteins As Biomarkers in Ovarian Cancer Article in Journal of Clinical Oncology. J Clin Oncol.

[231] Selamat SA, Chung BS, Girard L, Zhang W, Zhang Y, Campan M, Siegmund KD, Koss MN, Hagen JA, Lam WL (2012). Genome-scale analysis of DNA methylation in lung adenocarcinoma and integration with mRNA expression. Genome Res..

[232] Schillebeeckx M, Schrade A, Löbs AK, Pihlajoki M, Wilson DB, Mitra RD (2013). Laser capture microdissection–reduced representation bisulfite sequencing (LCM-RRBS) maps changes in DNA methylation associated with gonadectomy-induced adrenocortical neoplasia in the mouse. Nucleic Acids Res..

[233] Jovanovic B, Mayer IA, Mayer EL, Abramson VG, Bardia A, Sanders ME, Kuba MG, Estrada MV, Beeler JS, Shaver TM (2017). A randomized phase II neoadjuvant study of cisplatin, paclitaxel with or without everolimus in patients with stage II/III triple-negative breast cancer (TNBC): Responses and long-term outcome correlated with increased frequency of DNA damage response gene mutations, TNBC subtype, AR status, and Ki67. Clin Cancer Res..

[234] Chen T, Cao C, Zhang J, Streets A, Li T, Huang Y (2022). Histologically resolved multiomics enables precise molecular profiling of human intratumor heterogeneity. PLOS Biol..

[235] Krysan K, Tran LM, Grimes BS, Fishbein GA, Seki A, Gardner BK, Walser TC, Salehi-Rad R, Yanagawa J, Lee JM (2019). The immune contexture associates with the genomic landscape in lung adenomatous premalignancy. Cancer Res..

[236] Pöschel A, Beebe E, Kunz L, Amini P, Guscetti F, Malbon A, Markkanen E (2021). Identification of disease-promoting stromal components by comparative proteomic and transcriptomic profiling of canine mammary tumors using laser-capture microdissected FFPE tissue. Neoplasia.

[237] Nikfar, M., Mi, H., Gong, C., Kimko, H., and Popel, A.S. (2023). Quantifying Intratumoral Heterogeneity and Immunoarchitecture Generated In-Silico by a Spatial Quantitative Systems Pharmacology Model. Cancers 2023, Vol. 15, Page 2750 15, 2750. 10.3390/CANCERS15102750. 10.3390/cancers15102750 PMC10216176 37345087

[238] Bae S, Na KJ, Koh J, Lee DS, Choi H, Kim YT (2022). CellDART: cell type inference by domain adaptation of single-cell and spatial transcriptomic data. Nucleic Acids Res..

[239] Lee Y, Park JH, Oh S, Shin K, Sun J, Jung M, Lee C, Kim H, Chung JH, Moon KC (2022). Derivation of prognostic contextual histopathological features from whole-slide images of tumours via graph deep learning. Nat Biomed Eng..

[240] Failmezger H, Muralidhar S, Rullan A, de Andrea CE, Sahai E, Yuan Y (2020). Topological tumor graphs: A graph-based spatial model to infer stromal recruitment for immunosuppression in melanoma histology a C. Cancer Res..

